# A study on the status quo and influencing factors of science identity in physics among junior high school students

**DOI:** 10.1371/journal.pone.0358695

**Published:** 2026-10-13

**Authors:** Danfeng Sun, Yankun Lyu, Yuping Yang

**Affiliations:** 1 Northeast Normal University, Changchun, China; 2 Shaanxi Normal University, Xi'an, China; 3 Minzu University of China, Beijing, China; Nanjing University, CHINA

## Abstract

This study examines the status of science identity of physics among middle school students, adopting a mixed-methods approach combining survey data and qualitative interviews, thereby addressing a gap in research on science identity in physics within the transitional context from integrated to discipline-specific curricula in early middle school. Quantitative analyses, including descriptive statistics, cluster analysis, correlation analysis, t-tests, and MANOVA, examined students’ science identity status, patterns, and gender differences. Qualitative interviews were analyzed using reflexive thematic analysis to explore underlying factors. The findings indicate that students’ science identity is moderately positive overall but characterized by an internal imbalance. While interest in science classes emerges as the strongest dimension, science role recognition remains notably weak, revealing an interest–recognition gap. A substantial proportion of students cluster within a neutral identity category, suggesting that the transition from integrated science to subject-based curricula represents a critical and malleable stage in identity development. Gender differences are significant across all dimensions of science identity and become more pronounced at higher grade levels, particularly in learning competence, practical performance, and career interest. Qualitative evidence suggests that these patterns may be associated with increasing academic demands following the transition to discipline-specific curricula, shifts in evaluation approaches, and internalized gender stereotypes. Additionally, limited peer discourse, low perceived proximity to the scientist role, and insufficient exposure to authentic scientific practices further constrain identity formation. Teacher recognition is a critical factor, with female students showing heightened sensitivity to it.

## Introduction

In an era of rapid technological advancement, cultivating high-level innovative talent has become a global priority in science education reform. At the core of this mission lies the development of science identity—a multidimensional psychological construct that defines how individuals perceive themselves within the scientific community and how they are recognized by others [1,2]. Recently, science identity has been incorporated into the core assessment dimensions of the PISA 2025 science framework, offering a broader lens for examining students’ sustained engagement and long-term commitment to science beyond traditional cognitive indicators [3,4].

Despite increasing attention to this construct, a concerning “leaky pipeline” persists globally: students’ attitudes toward science often decline sharply during the transition from primary to secondary education [5]. This erosion is particularly pronounced in physics, a discipline frequently perceived as abstract and intellectually demanding [6]. For many learners, this period represents a critical turning point at which general interest in science either consolidates into a stable disciplinary identity or dissipates under escalating academic demands and shifting social pressures.

However, limited research has specifically examined the development and underlying mechanisms of physics identity during this transitional stage. This gap is especially salient within the Chinese educational context, where subject specialization begins in Grade 8. This population warrants empirical attention for two interrelated reasons. First, the curricular transition from integrated science in Grade 7 to discipline-specific physics in Grade 8 represents a structural feature unique to the Chinese educational system. Unlike gradual subject specialisation models common in Western contexts, this abrupt shift exposes students to a dramatically new learning environment characterised by increased abstraction, specialised terminology, and distinct problem-solving conventions—conditions that may trigger identity negotiation processes not captured by research conducted in gradual-transition settings. Second, Chinese junior high school students remain an under-researched population in the physics identity literature. The vast majority of existing studies have been conducted in Western educational contexts, leaving the applicability of established identity frameworks to East Asian educational systems—with their distinct cultural values, classroom norms, and examination pressures—largely untested. Addressing this gap is essential not only for theoretical generalisability but also for informing contextually appropriate educational interventions in non-Western settings.

Given this research gap, the present study poses the following research question: How does physics identity manifest among Chinese junior high school students during the transitional stage from integrated science to subject-specific physics instruction? Specifically, we seek to characterize the overall status of physics identity across grade levels and genders, and to identify distinct patterns of identity development that may exist among students during this transitional period.

Drawing on data from 1,009 students during this critical period, the present study addresses this gap by investigating how the onset of formal physics education shapes divergent trajectories of science identity—particularly in terms of gender differences—and by exploring the emergence of a “recognition gap” within this subject-specific transition. Through this lens, the study provides empirical evidence to inform educational interventions aimed at sustaining science identity as a foundational component of global scientific literacy.

## literature review

Tracing its origins to the psychological foundations of the social sciences, the construct of science identity draws heavily upon the developmental theory of Erik Homburger Erikson (1959/ 1980). In Identity *and the Life Cycle*, Erikson conceptualized identity as a dual-axis phenomenon encompassing both idiosyncratic self-perception and collective group identification. He proposed that a coherent identity manifests as a “sense of self-sameness and continuity,” linking individuals’ past experiences with their anticipated future trajectories (pp. 102–114). Crucially, Erikson emphasized that such internal coherence depends on recognition by significant others, highlighting identity as both a psychological achievement and a socially mediated process. This perspective provides a valuable analytical lens for examining recognition-related disparities in science learning contexts, particularly in physics education.

From a philosophical standpoint, identity refers to the affirmation and enactment of identity in relation to oneself and one’s social environment, originating in the enduring inquiry into “Who am I?”. This line of thought can be traced to René Descartes, whose proposition “Cogito, ergo sum” (“I think, therefore I am”) positions self-awareness as the foundation of one’s perceived capabilities and vocational orientations. Integrating these perspectives, identity can be understood as both an internally constructed and socially negotiated process.

Building on these theoretical foundations, science identity represents a domain-specific extension of identity, referring to how individuals perceive themselves in relation to science, evaluate their competence in scientific practices, and express their willingness to pursue science-related careers. Within contemporary educational discourse, this construct aligns closely with the dimension of scientific attitudes and responsibility in the national curriculum framework [7]. It plays a pivotal role in shaping students’ understanding of the nature of science, including their mastery of scientific knowledge and methods, their perception of the scientific community, and the development of scientific attitudes and social responsibility.

Existing scholarship consistently characterizes science identity as a multidimensional construct that has attracted sustained attention in science education research [8–10]. Empirical studies have demonstrated that science identity functions as a critical psychological mechanism influencing students’ engagement, persistence, and career aspirations [11,12]. Students who internalize a science identity are more likely to participate in optional science experiences and pursue STEM-related careers [13,14]. Beyond simple affiliation, science identity shapes whether individuals perceive themselves as “science persons,” thereby promoting greater agency, enhanced discursive participation, improved practical performance, and more sophisticated meaning-making in scientific contexts [11,15,16]. Following the recognition of its importance, research has increasingly focused on the influencing factors, theoretical models, and measurement approaches associated with science identity. A range of contextual variables—including gender, disciplinary learning experiences, informal science engagement, family background, and teacher evaluations—have been identified as significant contributors to its development [1,17]. In terms of theoretical modeling, Carlone and Johnson (2007) proposed a tripartite framework comprising competence, performance, and recognition, identifying recognition as a central mechanism in identity construction [1]. Competence refers to an individual’ s internalised belief in their ability to understand scientific knowledge and solve scientific problems—a self-referential judgment of capability; performance, by contrast, refers to the outward demonstration of scientific practices, including experimental skills, inquiry-based activities, and the application of knowledge to authentic contexts. Extending this model, Hazari et al. (2010) introduced interest as an additional dimension, demonstrating—based on a large-scale retrospective study of over 3,000 undergraduates—that secondary school physics experiences play a critical role in shaping science identity [6]. They conceptualised interest as a unified construct encompassing both enjoyment of science and career aspirations. In the Chinese educational context, however, career interest and classroom interest often diverge: students may enjoy physics classes without considering physics-related careers, or may aspire to scientific careers while finding classroom instruction uninspiring. Building upon Hazari et al.’s framework, the present study therefore treats these two facets as context-specific manifestations of the broader interest construct, differentiated by their temporal scope (short-term classroom enjoyment versus long-term career orientation) and contextual scope (formal learning environment versus future occupational imagination).

Within this framework, physics identity has emerged as a particularly salient and influential construct. Research indicates that it possesses cross-disciplinary predictive power, serving as an important indicator of both biological identity and overall science identity [18]. Measurement studies further support this position. Chen et al. (2022) developed validated instruments grounded in existing theoretical models and, based on a survey of 510 high school students, identified a notable paradox: although students report the lowest levels of identity in physics compared to chemistry and biology, physics identity exerts the strongest influence on their intention to pursue science-related university studies [11].

From a developmental perspective, early adolescence is widely recognized as a critical period for the formation of science identity. Empirical research suggests that around the age of 14 represents an optimal window for cultivating students’ interest in science and related career aspirations [19]. However, despite growing recognition of its importance, this stage remains insufficiently examined in empirical research. While previous studies have documented a significant decline in science identity from Grade 8 and Grade 10 [11], the full transitional phase between Grade 8 and Grade 9 has received limited scholarly attention. This gap is particularly salient in East Asian contexts, such as China, where students begin discipline-specific science learning from Grade 8. Within such systems, the potential identity shock associated with this curricular shift—and the underlying psychological and developmental processes—remains underexplored.

## Methodology

### Research Tools

The research instruments were adapted from the science identity questionnaire developed by Chen and Wei (2020), with additional modifications to incorporate a physics-specific perspective. The original instrument employed a Likert scale and comprised 23 items across four dimensions: scientific competence/self-efficacy, interest and performance in science classes, recognition as a science person, and interest in science-related careers. The modifications followed three principles: systematically replacing all general references to “science” with “physics”; translating “science person” as “little physics scientist” in the recognition dimension (Items 13–16) to better resonate with the local middle-school context; and making minor wording refinements to enhance clarity and emphasis (e.g., specifying “physics knowledge” in Item 9, adding “if I try my best” in Item 12). The fundamental theoretical basis and the core constructs under consideration—competence/self-efficacy, recognition, interest, and career aspiration—remained unchanged throughout the adaptation. To establish content validity, two physics education experts with extensive experience in secondary school physics teaching and research independently reviewed the adapted items against criteria of disciplinary relevance, clarity, and theoretical alignment. Through two rounds of review, the experts suggested revisions such as explicitly specifying “physics experiments” in Item 4 and simplifying technical expressions in Items 8 and 9, after which both reached consensus that all items were appropriately adapted. This item-level comparison and expert validation process ensures that all adaptations are justifiable and adequate for capturing physics-specific aspects of science identity.

After revising the measurement items of the questionnaire, we distributed 300 questionnaires and 277 valid samples were obtained. The reliability of the scale was tested using SPSS 29.0, and the validity of the revised scale was comprehensively evaluated using item discrimination test and validity analysis methods. The item discrimination test used the extreme group comparison method, divided into high and low groups (the upper and lower 27% of the total scores were used to define the high and low groups), and used t-test for difference comparison. The results showed that the high and low groups differed significantly on all 23 items (p < 0.05), meaning that all 23 items had good discrimination. The overall Cronbach's α value of the scale was 0.941, and the Cronbach's α value range after deleting items was between 0.937 and 0.940. The reliability coefficient did not increase significantly after deleting any item, so the items were not deleted and all items were retained. Exploratory factor analysis was conducted on the questionnaire, with a KMO value of 0.919. The data passed the Bartlett's sphericity test ($\chi^{\text{2}}$= 4233.496, p < 0.001), and the research data were suitable for factor analysis. The exploratory factor analysis was used to determine the factors, because the factors of the dimensions were correlated, the principal axis factor decomposition method and the optimal oblique rotation method were selected, and 4 factors were forced to be extracted. However, due to the modification of the measurement variables and the difference in the sample of the questionnaire from the original questionnaire's sample group, the factor item structure obtained was somewhat inconsistent with the original scale. We therefore re-conducted exploratory factor analysis using the eigenvalue-greater-than-1 criterion, and the results were divided into a five-factor model. The factor load coefficient of each item reached 0.4 or above, and the cumulative percentage of variance explained by the five factors reached 61.535%.

Again, 350 questionnaire data were collected, with 325 valid samples. Confirmatory factor analysis was conducted using Jamovi software. The model of 5 factors had the best fit. After fine-tuning the 5-factor model based on the MI value, the main indicators were as follows: $\chi^{\text{2}}$ (218, N = 325) = 556.118, $\chi^{\text{2}}$/df = 2.551 < 3, RMSEA = 0.069 < 0.08, SRMR = 0.053 < 0.1, p < 0.001, TLI = 0.918 and CFI = 0.929 were all greater than 0.9, and the overall model fit was good. For the discriminant validity analysis of HTMT values, all HTMT values were less than 0.85, and there was good discriminability between the factors. Therefore, the revised questionnaire was divided into five dimensions, and internal consistency analysis was conducted for each dimension. The reliability of each dimension was good, as shown in Table 1.

**Table 1:** Reliability of each dimension.

Dimension	*Cronbach’* α
Science Learning Competence Identity	0.889
Scientific Practice Performance Identity	0.710
Interest in scientific career	0.871
Interest in science class	0.869
Science Role Recognition	0.910

### Participants

This study adopted a random sampling method to select a total of three public junior high schools in two cities within Guangdong Province in China, with students from grades 8 and 9 as the survey subjects. The three schools were selected based on two criteria. First, to ensure sample diversity, we intentionally chose schools located in different geographic areas. Second, all selected schools are government-funded public institutions, which are the backbone of basic education in China and typically maintain relatively stable instructional quality while drawing from socioeconomically diverse student populations. We sampled students from both Grade 8 and Grade 9 within each school to minimize demographic differences between grade-level groups. The investigation was conducted from 10 April 2025–25 April 2025. All procedures were conducted after obtaining written informed consent. As the participants were minors, written informed consent was obtained from both their parents and school guardians prior to participation. The questionnaire was administered to participants in paper form. Before completion, teachers informed students of the purpose of the questionnaire, explaining that participation was voluntary, responses were anonymous, and the data would not be used for any form of student evaluation. A written explanation of the study was also provided at the beginning of the questionnaire. Students were informed that they could withdraw at any time without any consequences. For the random interview component, informed written consent was obtained from participants and their guardians prior to the interviews. Only those who provided consent were included. The interview questions did not involve personal privacy, and all data were collected anonymously with strict protection of confidentiality. This study has received ethical approval and the number is NENU20250327.

### Methods

A total of 1,100 questionnaires were distributed, and 1,009 valid responses were collected, yielding a response rate of 91.7%. Among them, 48.7% were male and 51.3% were female, with 55.4% from grade 8 and 44.6% from grade 9. The reliability coefficient α of this survey was 0.951, indicating high data reliability and quality, which can be used for further analysis. Bartlett’s test of sphericity was significant (p < 0.001), and the KMO measure of sampling adequacy was 0.947, indicating that the data were suitable for factor analysis. In the quantitative part of this study, descriptive statistics were computed to characterise the overall sample and to describe the features of different subgroups. Independent-samples t-tests were conducted to examine gender differences across the five dimensions of physics identity. A multivariate analysis of variance (MANOVA) was performed to test whether gender differences varied significantly across grade levels, as well as to examine the main effects of gender and grade. Pearson correlation analyses were used to examine the relationships between demographic variables (gender, grade) and each dimension of physics identity. Additionally, K-means cluster analysis was employed to explore whether distinct subgroups of students could be identified based on their patterns of physics identity during the transitional stage from integrated science to discipline-specific physics curricula in early middle school.To complement and contextualise the quantitative findings, semi-structured interviews were conducted with a purposive subsample of 18 students (10 female, 8 male) selected from the larger survey cohort. Sampling employed a combination of extreme-case and maximum-variation strategies, selecting participants based on their physics identity scores (top and bottom 15%) and disciplinary backgrounds to ensure diverse experiential representation.

Interviews were conducted by the first author using a protocol developed around key constructs from the quantitative analysis—namely, physics identity salience, self-perception of bodily appearance, and social comparisons in academic and daily contexts. Each session lasted 10–15 minutes, was audio-recorded, and transcribed verbatim. Data were analysed using reflexive thematic analysis [20]. The first author led the coding process, with a second independent coder analysing 30% of transcripts to establish reliability (intercoder agreement = 89%); discrepancies were resolved through consensus. Member checking with five participants confirmed the resonance of emerging themes. Direct quotations are presented in the Discussion to substantiate interpretive claims. The combination of quantitative and qualitative methods allows for a fuller picture of the state of physics identity.

### Measurement bias test

Since the same questionnaire was used for a one-time survey to collect data in this study, and the measurement items have a self-assessment tendency, issues such as artificial covariation among the subjects’ implicit theories and problems related to the common method variance may affect the collected data. To ensure the accuracy and reliability of the research results, procedural control for common method bias was carried out in the questionnaire compilation process, including anonymity guarantee, fear avoidance, randomization sequence, and avoidance of neutral expressions. After collecting the data, statistical tests were conducted according to Podsakoff’ s suggestions [21], using Harman's single-factor CFA to determine the severity of common method bias in the study. The results showed that multiple indicators of the single-factor model deviated significantly from the critical value (Table 2), and the indicators of the five-factor model were significantly better than those of the single-factor model. Therefore, common method bias will not have a serious impact on the results.

**Table 2:** Harman's single-factor CFA test and model comparison.

	$\chi^{2}$	df	CFI	TLI	RMSEA	RMSEA 90% CI		SRMR	AIC	BIC
						Lower	Upper			
Single factor	5526***	230	0.680	0.648	0.151	0.148	0.155	0.0813	55057	55396
Five factors	1451***	220	0.926	0.915	0.074	0.071	0.071	0.0745	51002	51391

**P < 0.01, ***P < 0.001.

### Data analysis

Based on the descriptive statistics of the questionnaire (Table 3), the overall science identity manifests a neutral-to-positive orientation. Significant variances were observed across different dimensions: students achieved the highest scores in Interest in Science Class, while Science Role Recognition received the lowest rating. The profile of the characteristics of science identity is shown in Fig 1.

**Table 3:** The overall situation.

Variables	*M*	*SD*
Science Learning Competence Identity	3.73	0.747
Scientific Practice Performance Identity	3.50	0.813
Interest in scientific career	3.13	0.979
Interest in science class	3.92	0.866
Science Role Recognition	2.67	0.999

**Fig 1:**
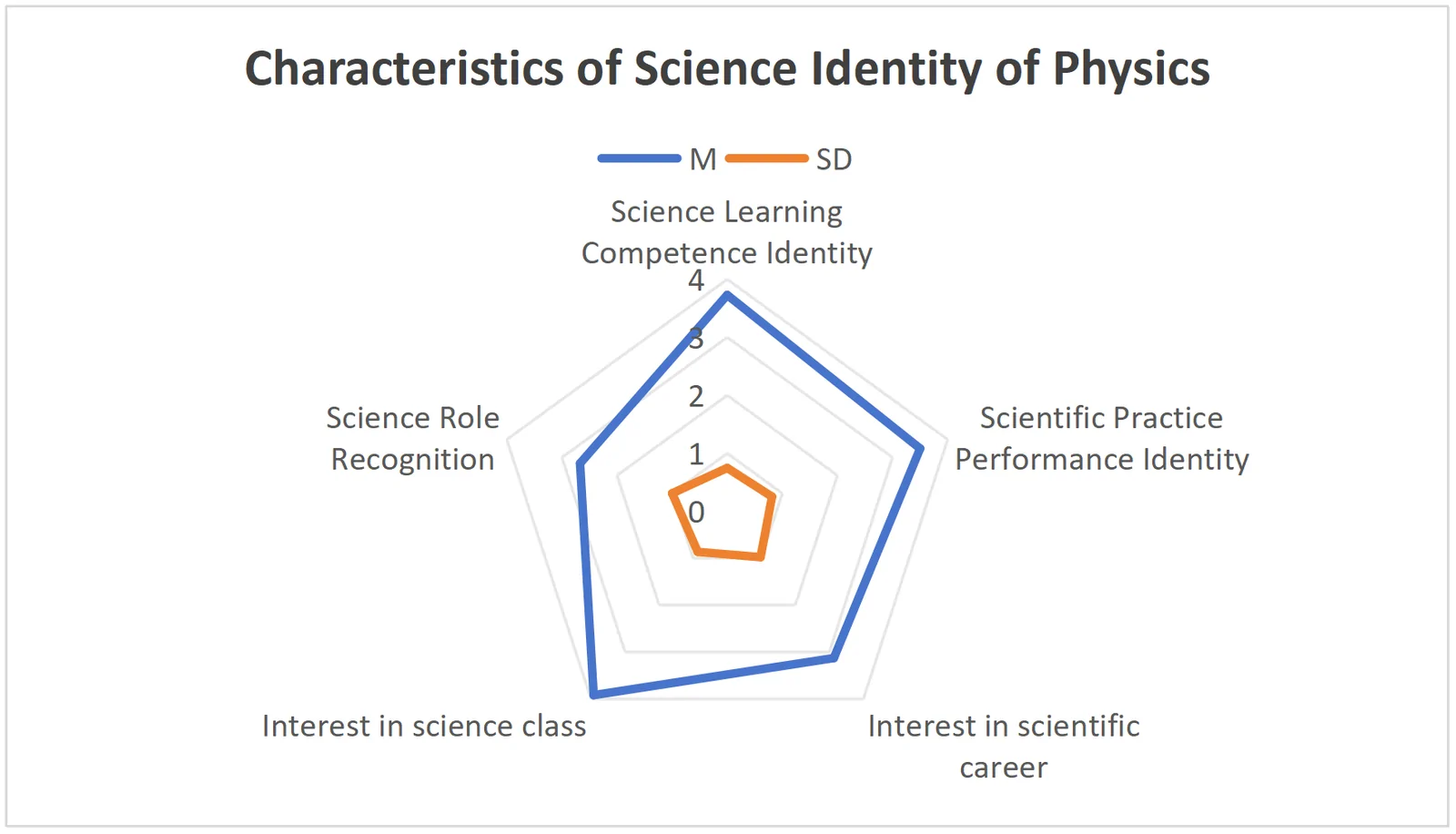
The characteristics of science identity.

A further analysis was conducted to examine variations in science identity across grade levels and gender. The results indicate that ninth-grade female students exhibit lower levels of science identity compared to eighth-grade female students, whereas ninth-grade male students demonstrate higher levels of science identity than their eighth-grade counterparts (Table 4).

**Table 4:** Descriptive statistics by grade level and gender.

	Male		Female		Total	
	M	SD	M	SD	M	SD
Grade 8	3.50	0.74	3.37	0.71	3.44	0.73
Grade 9	3.54	0.63	3.23	0.65	3.37	0.67

Given the observed gender differences in performance, male and female students were analyzed separately, and descriptive statistics were calculated across all dimensions. The results show that male students scored higher than female students on all dimensions. To examine whether the gender differences in physics identity varied significantly across grade levels, a multivariate analysis of variance (MANOVA) was conducted, with the five identity dimensions as dependent variables(see Table 5). Results revealed that the overall grade-by-gender interaction effect was not significant (Wilks’ Λ = 0.988, p = 0.073). Subsequent univariate analyses showed that significant interaction effects were observed for Science Learning Competence Identity, [F(1, 829) = 7.079, p = 0.008, $\eta^{\text{2}}$ = 0.008], and Interest in Science Class, [F(1, 829) = 4.147, p = 0.042, $\eta^{\text{2}}$ = 0.005], suggesting that the gender gap in these two dimensions may vary across grade levels. However, given the non-significant multivariate interaction, these univariate findings should be interpreted with caution.

**Table 5:** MANOVA: Grade and gender for the variables.

Variables	ANOVA results					
	Gender F(1, 825)	Partial$\;{\;\eta }^{2}$	Grade F(1, 825)	Partial$\;\eta^{2}$	Interaction F(1, 825)	Partial $\eta^{2}$
Science Learning Competence Identity	26.65^***^	0.031	21.71^***^	0.026	7.08^**^	0.008
Scientific Practice Performance Identity	14.08^***^	0.017	1.47	0.002	3.38	0.004
Interest in scientific career	21.60^***^	0.025	7.34^**^	0.009	2.62	0.003
Interest in science class	9.32^**^	0.011	0.76	0.001	4.15*	0.005
Science Role Recognition	7.64^*^	0.009	11.02^***^	0.013	0.30	0.000

Independent-samples t-tests were further conducted to examine whether gender exerted heterogeneous effects across these dimensions (Table 6). The findings indicate that all p-values are below 0.05, with the dimensions of science learning competence identity, scientific practical competence identity, and interest in science career reaching a significance level of p < 0.001. According to the guidelines proposed by Jacob Cohen [22], the effect size for science learning competence identity is at a medium level, while the effect sizes for the remaining dimensions are large. To control for the inflated familywise error rate associated with multiple comparisons, we applied the Bonferroni correction to the five independent-samples t-tests conducted across the five dimensions of physics identity. With a total of five comparisons, the corrected significance level was set at $\frac{\alpha }{n}$= 0.05 / 5 = 0.01. Accordingly, only results with p-values below 0.01 were considered statistically significant after correction. All five t-tests yielded p-values below 0.01 (two at p < 0.01 and three at p < 0.001), indicating that all gender differences remained statistically significant after the Bonferroni adjustment.

**Table 6:** T-test results.

Variables M = Male F = Female		Sample size (*N*)	Mean value (*M*)	Standard Deviation (*SD*)	*t*	95% Confidence Interval		Cohen’s d	95% Confidence Interval	
						Lower	Upper		Lower	Upper
Science Learning Competence Identity	M	471	3.87	0.73	5.58***	0.17	0.36	0.74	0.21	0.47
	F	505	3.60	0.74						
Scientific Practice Performance Identity	M	477	3.60	0.86	3.64***	0.09	0.29	0.81	0.10	0.36
	F	509	3.41	0.76						
Interest in scientific career	M	486	3.28	0.99	4.89***	0.18	0.42	0.97	0.19	0.45
	F	512	2.99	0.95						
Interest in science class	M	486	4.00	0.86	2.67**	0.04	0.25	0.87	0.03	0.30
	F	514	3.86	0.87						
Science Role Recognition	M	480	2.77	1.03	3.03**	0.07	0.31	0.99	0.08	0.34
	F	509	2.58	0.95						

**P < 0.01, ***P < 0.001.

To further categorize the sample, K-means cluster analysis was performed. To determine the optimal number of clusters, we employed the elbow method. The elbow plot showed that the reduction in within-cluster sum of squares levelled off after k = 3, indicating that k = 3 was the optimal solution. The K-means algorithm was then applied with k = 3 and converged on a three-cluster solution.. The first group, labeled as Positive Identity (Cluster 2), accounts for 29% of the total sample. The second group, Negative Identity (Cluster 3), comprises 19%. The final and largest group is the Neutral Identity (Cluster 1), constituting 52% of the participants. Overall, the majority of students belong to the neutral identity group, while the proportion of students with positive identification exceeds those with negative identification (Table 7).We further conducted cluster analyses separately by gender and grade, and found that the three clusters—high, moderate, and low identity—could also be distinguished within each subgroup, with profile patterns similar to those observed in the total sample.

**Table 7:** Final cluster centres for the five dimensions.

	Cluster 1 (n = 524)	Cluster 2 (n = 293)	Cluster 3 (n = 192)
Science Learning Competence	3.70	4.49	2.79
Scientific Practice Performance	3.46	4.25	2.58
Interest in Scientific Career	2.97	4.11	2.04
Interest in Science Class	3.91	4.69	2.78
Science Role Recognition	2.55	3.66	1.55

Correlations between Demographics and Identity Dimensions Correlation analysis (Table 8) reveals that Grade is negatively associated with “Science Role Recognition” and “Science Learning Competence Identity.” Males were coded as 1, females as 2; eighth-grade students were coded as 1, and ninth-grade students as 2. Notably, Gender displayed a significant negative correlation with all measured dimensions of science identity.

**Table 8:** Pearson correlation test.

	Science Learning Competence Identity	Scientific Practice Performance Identity	Interest in Scientific Career	Interest in Science Class	Science Role Recognition
Gender	−.176**	−.116**	−.153**	−.084**	−.096**
Grade	−.152**	.036	−.045	.056	−.074*

* *p* < 0.05 ** *p* < 0.01.

Analyzing the mean scores of individual items reveals that the top five highest-scoring items span across various identity dimensions (Table 9), yet all are closely associated with Science Learning Competence Identity and Interest in Science Class. Specifically, the item “I believe I can learn a lot of scientific knowledge in physics class” yielded the highest mean score. Conversely, among the five lowest-scoring items, four are situated within the Science Role Recognition dimension, while one originates from Interest in Scientific Career. These findings highlight a pronounced discrepancy between students’ academic confidence and their professional role identification. One-sample t-tests confirmed that both the five highest-scoring and the five lowest-scoring items were significantly different from the scale midpoint (3.0), all at p < .001. Subsequently, independent-samples t-tests were performed to examine gender differences across these ten items. The results showed that only three items—Q10, Q16, and Q17—did not differ significantly by gender, while all other items showed significant gender differences. Notably, for the item “My teacher thinks I am a little physicist”(Q15), a significant gender difference was observed(t(891) = 3.008, p = 0.003; Cohen’s d = 1.037, 95%CI [0.070, 0.333]), which could shed light on the recognition mechanisms discussed in the Discussion.

**Table 9:** Differences in item measures.

Top Five Scoring Indicators (in descending order)				Bottom Five Scoring Indicators (in ascending order)			
	t-value	Cohen’d	95%CI		t-value	Cohen’d	95%CI
Q10. Believe that one can learn a lot of scientific knowledge in the physics class. (D1)	37.31^***^	0.92	[1.16,1.33]	Q14. My classmates think I am a little physics scientist (D5)	−12.46^***^	1.07	[-0.48,-0.35]
Q3. Have the ability to complete physics homework. (D1)	41.35^***^	0.81	[1.29,1.47]	Q15. My teacher thinks I am a little physics scientist (D5)	−12.61^***^	1.04	[-0.49,-0.35]
Q17. Consider that the physics knowledge taught in class is very important in real life (D4)	32.86^***^	0.96	[1.01,1.18]	Q16. My family and friends think I am a little physics scientist (D5)	−7.55^***^	1.09	[-0.319,-0.186]
Q18. Like the scientific instruments and equipment in the physics class (D4)	28.49^***^	1.03	[0.87,1.03]	Q13. I think I am a little physics scientist (D5)	−6.46^***^	1.11	[-0.28,-0.15]
Q9. Be able to use physics knowledge to explain natural phenomena in daily life (D1)	27.86^***^	0.95	[0.851,1.01]	Q22. Plan to engage in a science career related to physics in the future (D3)	−3.54^***^	1.08	[-0.18,-0.05]

To complement and elaborate upon the quantitative results, semi-structured interviews were conducted with randomly selected students from Grades 8 and 9. Through an iterative process of coding and theme refinement, five overarching themes emerged from the interview data: (1) knowledge difficulty and identity, (2) lack of peer recognition, (3) dual construction of teacher recognition, (4) low career interest in physics, and (5) grade-level decline in competence beliefs. Each theme comprises several sub-themes that capture the nuanced ways in which students articulate their experiences (Table 10).

**Table 10:** Emergent Themes and Sub-themes.

Summary Table of Emergent Themes and Sub-Themes from Interview Findings	
Theme	Sub-Theme
1. Knowledge Difficulty and Identity	1.1 Grade 8 content is easier than Grade 9
	1.2 Novelty of a new subject
2. Lack of Peer Recognition	2.1 Limited peer interaction on the topic
	2.2 Perceived distance from scientists
	2.3 Lack of self-confidence
3. Dual Construction of Teacher Recognition	3.1 Academic performance
	3.2 Classroom performance
	3.3 Teacher feedback
4. Low Career Interest in Physics	4.1 Uncertainty about future plans
	4.2 Lack of knowledge about physics careers
	4.3 Limited extracurricular exposure
	4.4 Apprehension about physics careers
5. Grade-Level Decline in Competence Beliefs	5.1 Attribution to academic performance
	5.2 Classroom pacing and teaching methods
	5.3 Personal learning engagement

## Discussion

The findings indicate that students’ physics identity is generally neutral-to-positive but exhibits an internal imbalance. While interest in science classes is the strongest dimension, science role recognition remains weak, confirming the “interest–recognition gap” reported by Hazari et al. (2010). Within identity frameworks, recognition mediates the translation of competence and performance into a stable science identity [1]. The prevalence of students in the neutral category further suggests that early adolescence represents a malleable stage, sensitive to contextual influences [23,24]. This imbalance is closely linked to the transition from integrated science to discipline-specific physics. Integrated curricula emphasize broad participation and experiential engagement, facilitating recognition across diverse learners [25]. In contrast, physics instruction increases abstraction, formalization, and evaluative focus on procedural and symbolic skills, narrowing recognition to specific forms of achievement [26,27]. This identity re-evaluation may disadvantage students whose prior strengths were more visible in collaborative or exploratory contexts [24,28].

Gendered disparities are evident, with female students demonstrating lower identity levels across multiple dimensions, particularly in learning competence, practical performance, and career interest in Grade 9. This aligns with research showing that gender gaps widen when learning environments privilege male-typed epistemic practices [26,29]. Qualitative evidence (see Table 10) suggests that cognitive load, increased abstraction, and diminished novelty may contribute to female students’ sense of competence and belonging [27,28]. The observed patterns of gender differences in physics identity—particularly the decline among female students across grades—can be usefully interpreted through the lens of Eccles and Wigfield’s situated expectancy-value theory (SEVT) [30]. According to this framework, individuals’ achievement-related choices, persistence, and identity development are shaped by their expectancy beliefs and subjective task values, which are in turn influenced by contextual factors such as socializers’ beliefs, cultural norms, and the learning environment.

In the context of our findings, the grade-level transition from Grade 8 to Grade 9 introduces increased cognitive load, greater abstraction of content, and diminished novelty—factors that may negatively shape female students’ expectancy beliefs regarding physics. As suggested by our qualitative data, female students’ perceptions of greater task difficulty and their lower confidence in mastering physics content may contribute to their subjective task values and their identification with physics as a domain. This interpretation is consistent with SEVT’s prediction that competence beliefs and task values are dynamic and context-dependent, and that they are particularly susceptible to environmental cues such as teacher feedback and peer recognition. Our finding that female students scored significantly lower on “Science Role Recognition”—particularly in relation to teacher recognition—supports this view, suggesting that perceived social messages about who belongs in physics may differentially shape expectancy and value beliefs for male and female students. Additionally, normative comparisons with male peers and internalized stereotypes further constrain self-recognition, consistent with prior studies on gendered self-assessment in STEM [29,31]. Overall, the curricular transition represents a structurally significant moment for identity development, involving the reorganization of epistemic practices, evaluation standards, and recognition mechanisms. Without deliberate pedagogical strategies, this transition may disproportionately filter out female students. Interventions should focus on clarifying recognition processes, broadening definitions of competence, and supporting students in translating their abilities into stable and affirming physics identities [1,24]. The consistently low scores in the “Science Role Recognition” dimension further point to systemic barriers in identity construction. Qualitative data suggest that this deficit stems from three interrelated factors: limited peer discourse surrounding scientific identity, a perceived distance between students and the “scientist” archetype, and low self-efficacy even among high-performing students. These findings resonate with the “science-as-special” discourse [32], in which science is constructed as an exclusive domain reserved for a select few, thereby discouraging broader identification among students. In addition, students’ low interest in physics-related careers appears to be closely associated with limited exposure to authentic scientific practices and a lack of understanding of science-related professions. This finding is consistent with the Science Capital framework, which emphasizes the role of access to science-related resources and experiences in shaping students’ aspirations [24]. Without diverse opportunities to engage with science beyond the classroom, students’ career intentions remain underdeveloped and abstract.

Teacher recognition emerged as a pivotal mechanism in shaping students’ science identity, particularly within the transitional context from integrated science to discipline-specific physics instruction. Within identity-based frameworks, recognition is not merely evaluative but constitutive of identity itself, functioning alongside competence and performance as a core dimension through which students come to see themselves—and be seen by others—as legitimate participants in science [6,33]. However, the present findings extend this line of work by showing that the meaning and operation of recognition are not static; rather, they are reconfigured during key curricular transitions. Existing literature has established that classroom recognition is deeply embedded in interactional practices and institutional norms [2,34]. In integrated science settings, recognition tends to be more diffuse and inclusive, often grounded in participation, curiosity, and general engagement. By contrast, the shift to discipline-specific physics entails more formalized epistemic practices, including abstract reasoning, symbolic representation, and performance-based evaluation. As noted in prior research on disciplinary cultures, physics in particular has been characterized as emphasizing narrow forms of competence and canonical ways of thinking [26,27]. The current study builds on these insights by demonstrating how such shifts in evaluative criteria reshape students’ access to recognition.

Empirically, while male students’ perceptions of teacher recognition remained relatively stable, female students in Grade 9 reported a significant decline. This pattern is consistent with prior findings that gender disparities in STEM often widen over time, particularly in contexts where evaluative standards become more rigid and competitive [23,29]. Interview data further reveal that students construct recognition through multiple channels, including verbal feedback, nonverbal cues, and written evaluations, echoing earlier work highlighting the subtle and cumulative nature of teacher–student interactions [31,35]. Importantly, the findings also resonate with research on the recognition gap, in which students’ demonstrated competence is not equally acknowledged across groups. Male students in this study exhibited increasing confidence in learning physics, likely supported by both improved performance and alignment with dominant instructional norms. Such alignment enhances the visibility and legitimacy of their participation, reinforcing what Hazari et al. (2010) describe as a positive feedback loop between recognition and identity development [6]. In contrast, female students—despite comparable levels of effort—may experience diminished recognition due to a misalignment between their learning approaches and the valued forms of participation in physics classrooms. This reflects a broader pattern identified in the literature, where gendered expectations and stereotypes subtly shape both teacher perceptions and students’ self-evaluations.

By situating these dynamics within the transition from integrated to discipline-specific curricula, this study adds a new dimension to existing research. While prior studies have documented declines in science identity across adolescence [24,36], they have paid less attention to how institutional shifts in curriculum structure may act as catalysts for such changes. The present findings suggest that this transition may produce a form of identity dissonance, as students must renegotiate their sense of competence and belonging under new evaluative regimes. In this sense, the observed decline in female students’ perceived recognition can be understood not only as an outcome of individual or interpersonal factors but also as a structurally mediated process. From a pedagogical perspective, these results underscore the importance of critically examining the “hidden curriculum” that operates through everyday classroom interactions. Consistent with prior calls for more equitable STEM pedagogy [27,33], the findings highlight the need to diversify what counts as competence, make recognition more explicit and inclusive, and actively counteract implicit biases in teacher feedback and participation structures. Without such efforts, female students may interpret increasing academic difficulty and shifting expectations as evidence of diminished ability or lack of recognition. Given that evidence suggests female students are particularly sensitive to social evaluative cues [12,37,38], the consequences for science identity development may be disproportionately negative.

## Conclusion

This study investigated the science identity of Grade 8 and Grade 9 students during the critical transition from integrated science to discipline-specific physics instruction. The findings reveal that students’ science identity is moderately positive overall but marked by a clear internal imbalance. Interest in science classes emerges as the strongest dimension, whereas recognition of the scientist role remains weak, confirming an interest–recognition gap. A substantial proportion of students fell into the neutral identity cluster, highlighting the malleability of science identity during this key curricular transition.

Significant grade-level differences were observed across all dimensions, with female students exhibiting a marked decline in learning competence, practical performance, and career interest by Grade 9. Qualitative evidence suggests that these trends are associated with increased cognitive demands, reduced novelty, shifts in evaluation approaches, and internalized gender stereotypes. Limited peer discourse, low perceived proximity to the scientist role, and insufficient exposure to authentic scientific practices further constrain identity formation, while teacher recognition emerges as a critical factor influencing students’ perceived competence and sense of belonging.

By focusing specifically on this transitional stage, the study addresses a critical gap in research on science identity in physics education. The findings underscore the importance of competence-supportive pedagogy, targeted interventions during transitional stages, explicit recognition support in classroom interactions, and expanded access to authentic science experiences and role models to support equitable and sustained development of science identity among all students. Specifically, teachers can adopt tiered questioning that scaffolds students from recall to application, provide process-oriented feedback emphasising effort and strategy, and structure group work to ensure equitable participation in hands-on tasks. Given that female students perceive significantly lower teacher recognition, teachers should adopt inclusive practices such as acknowledging diverse forms of participation and using specific, growth-oriented praise that avoids artificiality, while displaying varied student work can signal that multiple ways of engaging with physics are valued. To ease the grade transition, schools should consider pre-transition orientation sessions, bridging modules linking concrete Grade 8 concepts to abstract Grade 9 content, and peer-mentoring programmes. Finally, to address low career interest, schools could invite former students in physics-related fields to share their career pathways, organise structured visits to science museums, and incorporate examples of contemporary Chinese female physicists into classroom instruction. When implemented in combination, these strategies may contribute to a more inclusive physics learning environment that sustains students’ science identity development throughout the middle school years.

## Limitations and future perspectives

Several limitations of the present study should be acknowledged. First, and of particular note, school-level differences were not explicitly controlled for in the primary analyses, as the principal objective was to examine overarching patterns rather than to conduct cross-school comparisons. Given the inherent nested structure of the data (students clustered within schools) and the limited number of schools involved, this limitation warrants particular consideration. Furthermore, in light of this nested structure, future investigations employing multilevel modeling with a larger sample of schools are recommended to more rigorously assess the effects of school-level variables on students’ science identity. Second, all data were collected via self-report questionnaires at a single time point, and common method bias was assessed solely through single-factor confirmatory factor analysis, without the incorporation of more robust techniques such as the marker variable approach. Although procedural precautions—including assurances of anonymity, counterbalanced item ordering, and clear, neutral wording—were implemented during questionnaire administration to minimize the risk of common method bias, these constraints are inherent to the cross-sectional design and cannot be entirely eliminated. Future studies should also consider alternative explanations, potential confounding factors, and contextual variables, such as socioeconomic status and school characteristics. Third, the present study primarily focused on delineating the current status of science identity and examining group differences, without exploring the dynamic processes underlying identity formation and change. Longitudinal designs are therefore recommended for future research to trace the developmental trajectories of science identity among the same cohort of students across different academic stages.
