# Supplementary material for: A study on the status quo and influencing factors of science identity in physics among junior high school students
Source: PLoS One. 2026 Oct 13;21(10):e0358695. doi: 10.1371/journal.pone.0358695 (PMC13660884; doi:10.1371/journal.pone.0358695)
Supplement: Supplementary file 1 [file pone.0358695.s002.docx]

**Response to Reviewers**

**We sincerely appreciate the constructive and insightful comments and suggestions from the anonymous reviewers, which have greatly helped us improve the quality and clarity of this manuscript. We have carefully addressed all the raised points point‑by‑point. Major revisions in the revised manuscript are highlighted using red color / track‑change mode. Below are our detailed responses to each comment.**

**Reviewer #1:**

**Comment 1.1**

**Reviewer:**

• On page 11, lines 8-12, the authors state that the research tools were "based on the scientific identity identification questionnaire developed by Sitong Chen et al." and that they "added a perspective of the physical science." However, the description of how the original instrument was adapted for physics is insufficient. The authors do not specify which items were modified, how the modifications were made, or whether content validity was established through expert review. While the subsequent psychometric testing (exploratory and confirmatory factor analyses) is thorough, the absence of a clear item-level description of the adaptation process raises questions about whether the instrument adequately captures physics-specific aspects of science identity. I would recommend that the authors provide a supplementary table comparing original and modified items, along with a description of the expert validation process.

**Response:**

**We thank the reviewer for the insightful comments. To ensure transparency in the instrument adaptation process, we provide a comparison table (see Table1) that presents the original items from Chen et al.‘s science identity questionnaire alongside our revised physics-specific version. The modifications followed three principles: systematically replacing all general references to "science" with “physics”(e.g., “science classes”→ “physics class”), translating "science person” as “little physics scientist”) in the recognition dimension (Items 13–16) to better resonate with the local middle-school context, and making minor wording refinements to enhance clarity and emphasis (e.g., specifying “physics knowledge” in Item 9, adding “if I try my best” in Item 12). The fundamental theoretical basis and the core constructs under consideration—competence/self-efficacy, recognition, interest, and career aspiration—remained unchanged throughout the adaptation. To establish content validity, two physics education experts, both experienced in secondary school physics teaching and research, independently reviewed the adapted items against criteria of disciplinary relevance, clarity, and theoretical alignment. Through two rounds of review, the experts suggested revisions such as explicitly specifying “physics experiments” in Item 4 and simplifying technical expressions in Items 8 and 9, after which both reached consensus that all items were appropriately adapted. This item-level comparison and expert validation process ensures that all adaptations are traceable, justifiable, and adequate for capturing physics-specific aspects of science identity.**

**The revisions have been made on pages 7-8 of the revised manuscript, and the changes are marked in red for easy identification.(“The research instruments...of science identity.”)**

**Table 1**

| Comparison Table: Original Science Identity Chart vs. Adapted Questionnaire | | | |
| --- | --- | --- | --- |
| Item | Original (Science Identity Chart) | Adapted (Physics Questionnaire) | Type of Change |
| 1 | I think I did well in science classes. | I think I did well in physics class. | Subject-specific (Science → Physics) |
| 2 | I am able to get a good grade in science subjects. | I am able to get a good grade in physics. | Subject-specific (Science → Physics) |
| 3 | I am able to complete my science homework. | I am able to complete my physics homework. | Subject-specific (Science → Physics) |
| 4 | I am proficient in using tools and operating apparatus in experiments. | I am proficient in using tools and operating apparatus in physics experiments. | Subject-specific (Science → Physics) |
| 5 | I can smoothly conduct a science inquiry activity. | I can smoothly conduct a physics inquiry activity. | Subject-specific (Science → Physics) |
| 6 | I can get a good grade in science and technology competitions. | I can get a good grade in physics and technology competitions. | Subject-specific (Science → Physics) |
| 7 | I think I am good at science. | I think I am good at physics. | Subject-specific (Science → Physics) |
| 8 | I can understand scientific laws and principles well. | I can understand physics laws and principles well. | Subject-specific (Science → Physics) |
| 9 | I am able to use science to explain the natural phenomena in daily life. | I am able to use physics knowledge to explain natural phenomena in daily life. | Subject-specific & Clarified (added "knowledge") |
| 10 | I believe I can learn a lot of knowledge in science classes. | I believe I can learn a lot of knowledge in physics class. | Subject-specific (Science → Physics) |
| 11 | I believe I will do well in science. | I believe I will be successful in physics. | Subject-specific (Science → Physics) & Phrasing |
| 12 | I believe I can learn even the hardest parts of scientific knowledge if I try. | I believe that if I try my best, I can learn the hardest parts of physics knowledge. | Subject-specific (Science → Physics) & Phrasing |
| 13 | I think of myself as a science person. | I think of myself as a little physics scientist. | Subject-specific & Emphasis (added "little") |
| 14 | My classmates recognize me as a science person. | My classmates recognize me as a little physics scientist. | Subject-specific & Emphasis (added "little") |
| 15 | My science teachers recognize me as a science person. | My physics teacher recognizes me as a little physics scientist. | Subject-specific (teacher) & Emphasis |
| 16 | My family and friends recognize me as a science person. | My family and friends recognize me as a little physics scientist. | Subject-specific & Emphasis (added "little") |
| 17 | I will learn more about science knowledge through a variety of sources. | I will learn physics knowledge through a variety of sources. | Subject-specific (Science → Physics) & Phrasing |
| 18 | I think the science knowledge taught in my classes is important in the real world. | I think the physics knowledge taught in class is very important in real life. | Subject-specific (Science → Physics) & Phrasing |
| 19 | I like the science equipment in my science classes. | I like the physics equipment and instruments in physics class. | Subject-specific (Science → Physics) & Expanded |
| 20 | I like to attend classes that are related to science. | I like to attend courses related to physics. | Subject-specific (Science → Physics) |
| 21 | I am interested in careers that are related to science. | I am interested in physics-related careers. | Subject-specific (Science → Physics) |
| 22 | I plan to pursue science careers in the future. | I plan to pursue a physics-related career in the future. | Subject-specific (Science → Physics) |
| 23 | I would feel comfortable talking to people who work in science careers. | I feel comfortable talking to people who work in physics-related careers. | Subject-specific (Science → Physics) |

**Comment 1.2**

• On page 13, lines 24-28, the authors indicate that three public junior high schools in two cities within Guangdong Province were selected using random sampling. However, the description of the sampling procedure is incomplete. The statement that "Each school was located in a different urban area or town and street with varying economic conditions" is vague. I would like to know what specific criteria were used to select these schools and whether the authors considered socioeconomic stratification in their sampling design. Additionally, the sample includes students from both Grade 8 and Grade 9 within each school, but the authors do not explain whether they controlled for school-level differences in their analyses. Given that only three schools were included, the extent to which these findings can be generalized to the broader Chinese junior high school population is unclear. I suggest that the authors acknowledge this limitation more explicitly in the Discussion section and consider whether a multilevel modeling approach might be more appropriate for their data structure.

**Response:**

**We thank the reviewer for the insightful and thorough comments. Regarding the school selection criteria, we have now provided a more detailed description in the revised manuscript. The three schools were selected based on two primary criteria. First, to ensure sample diversity, we intentionally chose schools located in different geographic and administrative contexts—specifically, one urban school and one suburban school in Dongguan City, and one urban school in Guangzhou City—to capture a broad range of student backgrounds across different urbanization levels. Second, all selected schools are government-funded public institutions, which represent the mainstream force of basic education in China and typically maintain relatively stable instructional quality while drawing from socioeconomically diverse student populations. Students from both Grade 8 and Grade 9 were recruited from each school. Since the primary aim of this study was to examine the overall patterns of science identity among junior high school students, rather than to compare school-level differences, we treated all participants as a single analytical sample.The revisions have been made on pages 10 of the revised manuscript, and the changes are marked in red for easy identification. (“The three schools were selected...populations.”)**

**Regarding the treatment of school-level differences, we acknowledge that we did not explicitly control for such differences in our original analyses, as our primary focus was on examining the overall patterns of science identity. We also recognize, as the reviewer suggests, that multilevel modeling would be a smarter analytical approach for nested data structures. In the present study, however, given our exploratory purpose and the relatively small number of schools, we retained the current analytical framework. But we have now included a recommendation in Limitations and Future Perspectives section for future research to employ multilevel modeling with a larger sample of schools to further examine school-level effects.The revisions have been made on pages 27 of the manuscript, and the changes are marked in red for easy identification. (“Moreover, given the nested nature of the data ... on students' science identity.”)**

**Regarding generalizability, we agree with the reviewer that the inclusion of only three schools, all within a single province, limits the extent to which our findings can be generalized to the broader Chinese junior high school population. In the Limitations and Future Perspectives section, we have explicitly acknowledged this limitation and have tempered our claims accordingly. We now state clearly that while our sample captures regional diversity within Guangdong Province, caution should be exercised when extending our conclusions to other provinces or national contexts. We have also suggested that future studies with larger, more geographically representative samples are needed to validate and extend our findings.The revisions have been made on pages 27 of the manuscript, and the changes are marked in red for easy identification. (“The three schools were selected...validate our conclusions.”)**

**Comment 1.3**

• The authors state on page 14, lines 5-7, that "interviews were conducted with the samples based on the results and phenomena of data analysis," but they provide no details about the qualitative component. How many students were interviewed? What was the sampling strategy for selecting interview participants? What interview protocol was used? How were the interview data analyzed (e.g., thematic analysis, grounded theory)? Who conducted the interviews, and what measures were taken to ensure reliability in coding? Without this information, it is difficult to evaluate the credibility of the qualitative evidence cited throughout the Discussion. For instance, on page 20, the authors claim that "qualitative evidence suggests that cognitive load, increased abstraction, and diminished novelty exacerbate stress" in female students, but no supporting quotes or coding schemes are presented to substantiate this claim.

**Response:**

**We thank the reviewer for the insightful and valuable comments. We have substantially expanded the Methods section to include a comprehensive description of the qualitative procedures. Specifically, we have now added(The revisions have been made on page 11-12 of the revised manuscript, and the changes are marked in red for easy identification. “Interviews were conducted...interpretive claims.”):**

**(1)The number of students interviewed (N = 18), with a breakdown by gender and academic year. (2)The sampling strategy: participants were purposively selected from the larger quantitative sample to represent maximum variation in academic performance and discipline. (3)The semi-structured interview protocol, which is now included as supplementary material (see Table2). (4)The analytical approach: we employed reflexive thematic analysis , with an inductive, data-driven coding process.Interviewer information: all interviews were conducted by the first author, who had completed graduate-level training in qualitative methods. To ensure reliability, two independent coders performed initial coding on 30% of the transcripts, with intercoder agreement reaching 89%; discrepancies were resolved through consensus discussion.**

**The Results section has been supplemented with a table presenting the themes and sub-themes that emerged from the interview analysis in the revised manuscript. However, in the table below(Table 3), representative participant quotes and the corresponding interview question numbers are additionally listed.**

**Table 2**

**Semi-Structured Interview Protocol for Physics Identity (Grades 8 & 9)**

**I. Opening and Background (1 question)**

**Q1. How would you describe your overall experience with physics learning so far? What comes to mind when you think about your physics classes?**

**II. Core Dimension: Science Recognition (3 questions)**

**Q2. Do you think your classmates see you as a “physics person” or someone who is good at physics? Why do you feel that way? Do your peers usually talk about things like this?**

**Q3. Do you think your physics teacher sees you as someone who is good at physics? What makes you feel that way? (Prompts: teacher's praise/criticism, classroom questioning, feedback on homework, attention in class, etc.)**

**Q4. How do your family members view your physics learning? Do they think you are good at physics?**

**III. Core Dimension: Competence Beliefs (2 questions)**

**Q5. How do you perceive the difficulty of physics content across different grades? Do you feel there is a difference between what you learned last year (or this year) and the other grade level? How has this affected your confidence in learning physics well?**

**Q6. Some people think that students who are good at physics tend to be boys rather than girls. What do you think of this view? What do you observe among your peers at your grade level?**

**IV. Core Dimension: Interest and Career Aspirations (2 questions)**

**Q7. Have you ever thought about pursuing a career related to physics in the future? What do you think physicists actually do? How much do you know about these careers? Have you participated in any physics-related extracurricular activities or visits (e.g., science museums)?**

**Q8. If you were to use one word or one sentence to describe your relationship with physics, what would it be? What experiences have shaped this feeling?**

**Table 3**

| **Summary Table of Emergent Themes and Sub-Themes from Interview Findings** | | | |
| --- | --- | --- | --- |
| **Theme** | **Sub-Theme** | **Representative Quotes** | **Corresponding Questions** |
| **1. Knowledge Difficulty and Identity** | **1.1 Grade 8 content is easier than Grade 9** | **"The content in Grade 8 is easier than that in Grade 9" (P1–P4)** | **Q1, Q5** |
|  | **1.2 Novelty of a new subject** | **"For Grade 8 students, physics is a new subject, so they feel better about it" (P4)** | **Q1, Q5** |
| **2. Lack of Peer Recognition** | **2.1 Limited peer interaction on the topic** | **"I don't know what my classmates think" / "We don't discuss who looks like a physicist" (P2, P3, P5)** | **Q2** |
|  | **2.2 Perceived distance from scientists** | **"I feel that scientists are far away from us—it's not realistic" (P5)** | **Q2** |
|  | **2.3 Lack of self-confidence** | **"Even with good grades, I'm not confident to say I look like a scientist" (P5)** | **Q2** |
| **3.Dual Construction of Teacher Recognition** | **3.1 Academic performance** | **"My grades" (P11)** | **Q3** |
|  | **3.2 Classroom performance** | **"My performance in physics class" (P11)** | **Q3** |
|  | **3.3 Teacher feedback** | **"The teacher's criticism and praise" / "Comments on my homework" (P11)** | **Q3** |
| **4. Low Career Interest in Physics** | **4.1 Uncertainty about future plans** | **"I don't know what I want to do in the future yet" (P6, P7, P9)** | **Q7** |
|  | **4.2 Lack of knowledge about physics careers** | **"I don't know what physics-related careers exist" (P5, P7)** | **Q7** |
|  | **4.3 Limited extracurricular exposure** | **"We have almost no physics-related activities—I've only been to the science museum" (P5)** | **Q7** |
|  | **4.4 Apprehension about physics careers** | **"Physicists have to think a lot—it's tiring" (P6) / "The requirements are very high" (P5)** | **Q7** |
| **5. Grade-Level Decline in Competence Beliefs** | **5.1 Attribution to academic performance** | **"It's related to grades" / "Boys in Grade 9 get better grades than girls" (P8, P9, P11)** | **Q5, Q6** |
|  | **5.2 Classroom pacing and teaching methods** | **"Grade 9 feels like more content and more lecturing" / "It has to do with the teacher's way of teaching" (P8)** | **Q5, Q6** |
|  | **5.3 Personal learning engagement** | **"I became more serious in Grade 9—I listened more and understood better" (P9)** | **Q5** |

**Comment 1.4**

• The authors report on pages 14-15 that they conducted Harman's single-factor CFA to test for common method bias. While the results suggest that common method bias is not a serious threat, I note that the authors used a single-factor CFA rather than the more commonly recommended Harman's single-factor test (which involves factor analysis of all items without rotation). More importantly, the authors did not employ more robust approaches to control for common method variance, such as the marker variable technique or the unmeasured latent method factor approach. Given that all data were collected via self-report questionnaires at a single time point, I would encourage the authors to discuss this limitation more transparently.

**Response:**

**We thank the reviewer for the detailed** **comments. For the assessment of common method bias, Harman's single-factor test is indeed the most commonly used approach. However, we chose the single-factor CFA primarily because it is theoretically more sensitive and accurate. We have discussed the limitation that all data were collected via self-report questionnaires at a single time point in the Limitations and Future Directions section. (The revisions have been made on page 27 of the revised manuscript, “Third, all data were collected via...design of this study.”)**

**Comment 1.5**

• On pages 8-9, the authors draw on Erikson' s developmental theory, Descartes' philosophical perspective, and Carlone and Johnson's (2007) tripartite framework. While this demonstrates breadth of scholarship, the integration of these disparate theoretical traditions is somewhat disjointed. The transition from Erikson's psychosocial theory to Descartes' epistemological philosophy is abrupt, and the connection to the contemporary science identity framework (competence, performance, recognition) is not clearly articulated. I would recommend that the authors strengthen the theoretical coherence by explicitly stating how these different perspectives collectively inform their conceptualization of science identity and their interpretation of findings. For example, how does Erikson' s notion of recognition by significant others translate into the operationalization of the "Science Role Recognition" dimension in their instrument?

**Response:**

**We appreciate the reviewer's comment. In our conceptualisation, we view Erikson's psychosocial theory, Descartes' philosophical view, and Carlone and Johnson's (2007) framework as operating at distinct but interconnected levels: Descartes' epistemology provides the philosophical foundation for identity as reflexive self-awareness; Erikson's theory supplies the social-developmental mechanism through which recognition from significant others shapes identity; and Carlone and Johnson's framework operationalises these insights into the three measurable dimensions of competence, performance, and recognition. Regarding the translation of Erikson's notion of recognition into our instrument, Erikson's emphasis on validation from significant others directly informed our operationalisation of the "Science Role Recognition" dimension through three social referents: peers, teachers, and family members. Additionally, since we are using a well-established scale rather than developing a new one based on theory, and no changes were made to the core ideas of the original scale, we have reinforced the theoretical perspective in interpreting the findings within the Discussion section in response to the reviewer's comments. Given that the original scale is grounded in a robust theoretical framework established by its original authors (Chen et al.), we believe that a detailed discussion of how the theory was translated into measurement items is beyond the scope of this study.**

**Comment 1.6**

• On page 12, the authors describe their revised five-factor model: Science Learning Competence Identity, Scientific Practice Performance Identity, Interest in Scientific Career, Interest in Science Class, and Science Role Recognition. However, the distinction between "Science Learning Competence Identity" and "Scientific Practice Performance Identity" is not adequately explained. Drawing on Carlone and Johnson's (2007) framework, competence and performance are distinct but related constructs—competence refers to one's internal sense of ability, while performance refers to the outward demonstration of science-related practices. The authors would benefit from explicitly discussing this distinction and justifying why they treated these as separate factors rather than as subdimensions of a broader competence-performance construct. Additionally, the inclusion of both "Interest in Scientific Career" and "Interest in Science Class" as separate dimensions is well-justified, but the authors should clarify how these relate to the "interest" dimension in Hazari et al.'s (2010) model, which conceptualizes interest as a unified construct that includes both enjoyment of science and career aspirations.

**Response:**

**We thank the reviewer for the insightful and thorough comments. We fully agree that the conceptual boundaries between these dimensions, including "Science Learning Competence Identity" and "Scientific Practice Performance Identity," required clearer articulation. So we have revised the manuscript to explicitly discuss the distinction between these two dimensions. (The revisions have been made on page 6 of the revised manuscript, “Competence refers ... authentic contexts.”)Drawing on Carlone and Johnson's (2007) original formulation, we now clarify that competence refers to an individual's internal sense of ability and understanding in relation to scientific knowledge, whereas performance refers to the outward demonstration or enactment of scientific practices, including inquiry skills, experimental manipulation, and application of knowledge in authentic contexts. This distinction is particularly meaningful in the Chinese secondary school context, where physics instruction typically involves both content mastery (e.g., understanding laws and principles) and practical performance (e.g., conducting experiments, solving real-world problems).**

**Regarding the two interest dimensions that Hazari et al. (2010) conceptualises interest as a unified construct encompassing both enjoyment of science and career aspirations. In the Chinese educational context, career interest and classroom interest often diverge: students may enjoy science classes (or particular topics within them) without considering physics-related careers, or conversely, may aspire to scientific careers while finding classroom instruction uninspiring. The factor analysis supported this empirical separation, Specifically, we view them as context-specific manifestations of the broader interest construct, differentiated by the temporal (short-term classroom enjoyment vs. long-term career orientation) and contextual (formal learning environment vs. future occupational imagination) scope of each.(The revisions have been made on page 6 of the revised manuscript, “They conceptualised interest ... occupational imagination”)**

**Comment 1.7**

• On page 17, the authors report using K-means cluster analysis to identify three subgroups: Positive Identity (29.44%), Negative Identity (19.62%), and Neutral Identity (50.94%). However, the authors do not specify how they determined the number of clusters (k=3). Was this determined empirically using methods such as the elbow method or silhouette analysis? Or was it theoretically justified? Without this information, the choice of three clusters appears somewhat arbitrary. Furthermore, the authors do not provide the cluster centroids or the variables used in the clustering procedure, making it difficult to interpret what distinguishes these three groups substantively. I would recommend that the authors include a table with cluster centers for each dimension and discuss whether the cluster solution was stable across different subsamples (e.g., by grade or gender).

**Response:**

**We thank the reviewer for the valuable comment. To identify distinct subgroups of students based on their patterns of physics identity, K-means cluster analysis was conducted using the five factor scores as clustering variables. We have added the table to the manuscript in accordance with the reviewer's suggestion(The revisions have been made on page 17 of the revised manuscript, “To determine ... the total sample”). To determine the optimal number of clusters, we employed the elbow method. The elbow plot showed that the reduction in within-cluster sum of squares levelled off after k=3, indicating that k=3 was the optimal solution. The K-means algorithm was then applied with k=3 and converged on a three-cluster solution.We further conducted cluster analyses separately by gender and grade, and found that the three clusters—high, moderate, and low identity—could also be distinguished within each subgroup, with similar profile patterns to those observed in the total sample.**

**Comment 1.8**

• On pages 16-17, the authors report that ninth-grade female students exhibit lower levels of science identity than eighth-grade female students, whereas ninth-grade male students demonstrate higher levels than their eighth-grade counterparts. This is a potentially important finding, but the authors present only descriptive statistics (Table 4) without reporting inferential tests for the grade-by-gender interaction. A two-way ANOVA or MANOVA would be appropriate to test whether the gender difference varies significantly by grade level. The authors should also consider reporting effect sizes for the interaction. Additionally, the t-test results in Table 5 show significant gender differences across all dimensions, but the authors use a Bonferroni correction or other adjustment for multiple comparisons? With five separate t-tests, the familywise error rate is inflated. I note that the authors apply Cohen's guidelines to interpret effect sizes, but they do not report the raw mean differences or 95% confidence intervals for the differences, which would provide a more complete picture of the magnitude of gender disparities.

**Response:**

**We thank the reviewer for the insightful and valuable suggestions. Following the reviewer's advice, we conducted a MANOVA to examine whether gender differences varied significantly across grade levels. Multivariate Tests showed that the overall grade-by-gender interaction effect was not significant (Wilks' Λ = 0.988, p = .073), indicating that, across the five dimensions collectively, the gender gap in physics identity did not change significantly with grade level. Although univariate analyses suggested potential interaction trends in the dimensions of Science Learning Competence Identity and Interest in Science Class, these results should be interpreted with caution given the non-significant multivariate interaction. We have now included the results table and provided relevant discussion in the revised manuscript. In addition, we followed the reviewer's suggestion to apply Bonferroni correction to the t-tests and have also supplemented the confidence intervals for the effect sizes.(The revisions have been made on page 14-16 of the revised manuscript, “To examine ... with caution”; “To control...Bonferroni adjustment”)**

**Comment 1.9**

• On page 18, the correlation analysis (Table 6) shows that grade is negatively associated with "Science Role Recognition" and "Science Learning Competence Identity." The authors state that this "underscore[s] the systemic influence of grade-level transitions," but the cross-sectional design precludes causal inference. It is equally plausible that pre-existing cohort differences (rather than the transition experience itself) account for the grade-level differences. The authors should temper their language and acknowledge this limitation more explicitly. They should also consider whether the correlation between grade and science identity dimensions is linear or whether there is a threshold effect, perhaps by examining the data using visual inspection or polynomial regression.

**Response:**

**We thank the reviewer for this important methodological observation regarding the interpretation of grade-level differences. We fully agree that our cross-sectional design precludes causal inference regarding the effects of grade-level transitions, and we acknowledge that pre-existing cohort differences may equally account for the observed grade-level patterns. We have revised the language throughout the manuscript to reflect this limitation, replacing causal language with more cautious descriptive terms (e.g., "associated with" rather than "influenced by") or just removing .We agree that exploring potential threshold effects would be an interesting avenue for future research.Given that grade in our study is a dichotomous variable with only two levels (Grade 8 and Grade 9), it is not appropriate to conduct polynomial regression analysis, as such analyses require at least three ordered levels to reliably detect nonlinear trends or breakpoints. With only two points, the relationship can only be described as linear Comment. However, we agree with the reviewers' opinion that future longitudinal studies could examine threshold effects, which would be a highly meaningful research endeavor. We discussion this in the Limitations and Future Perspectives section in the revised manuscript.The revisions have been made on pages 27 of the manuscript, and the changes are marked in red for easy identification.（“Moreover, given the nested nature of the data ... on students’ science identity.”)**

**Comment 1.10**

• On pages 18-19, the authors present the top five and bottom five scoring items (Table 7). The observation that four of the five lowest-scoring items are in the "Science Role Recognition" dimension is illuminating. However, the authors do not report the standard deviations or effect sizes for these item-level differences. It would be valuable to know whether the item means differ significantly from the scale midpoint (3.0) and whether there are grade or gender differences on specific items. For example, the item "My teacher thinks I am a little physics scientist" (Q15) might have distinct patterns for male and female students that could shed light on the recognition mechanisms the authors discuss in the Discussion.

**Response:**

**We thank the reviewer for this valuable suggestion regarding the item-level analysis. Following the reviewer' s advice, we conducted additional analyses to further examine the patterns of the lowest-scoring items and top-scoring items.**

**First, we performed one-sample t-tests to determine whether the mean scores of each of these ten items differed significantly from the scale midpoint (3.0). The results revealed that all ten items differed significantly from the midpoint (all p < .001), confirming that students' ratings on these recognition-related items were consistently and significantly lower or higher than the neutral level.**

**Second, we conducted independent-samples t-tests by gender for each of the ten items. Notably, for the item "My teacher thinks I am a little physicist" (Q15)—which the reviewer specifically highlighted—we found a significant gender difference: male students scored significantly higher than female students on this item, suggesting that male students perceive greater recognition from their teachers than their female counterparts. This finding provides important empirical support for the recognition mechanisms discussed in our manuscript, as it indicates that teacher recognition is not uniformly experienced across genders and may contribute to the observed gender gap in science identity.We have now incorporated these additional results into the revised manuscript, including a new table reporting the one-sample t-test results against the midpoint(table 8 in the revised manuscript) , and the statement of gender comparison results. We believe these analyses substantially strengthen the empirical grounding of our discussion on recognition mechanisms.(The revisions have been made on page 18-19 of the revised manuscript, “ In addition, we conducted ... teachers”)**

**Comment 1.11**

• Throughout the Discussion, the authors make causal claims that are not warranted by their cross-sectional data. For example, on page 20, they state: "Qualitative evidence suggests that cognitive load, increased abstraction, and diminished novelty exacerbate stress and undermine female students' sense of competence and belonging." While this may be a plausible interpretation, the authors have not established temporal precedence or ruled out alternative explanations. I would recommend using more cautious language (e.g., "is associated with," "may contribute to," "suggests a relationship") and acknowledging that longitudinal data would be needed to test causal hypotheses.

**Response:**

**We thank the reviewer for this important methodological observation. We fully agree that our cross-sectional data do not warrant causal claims, and we appreciate the opportunity to revise our language to more accurately reflect the scope of our findings.We have systematically reviewed the entire Discussion section and revised all causal language accordingly. Specifically, we have replaced terms such as “exacerbate,” “undermine,” and “lead to” with more cautious expressions including “is associated with,” “may contribute to,” “suggests a relationship,” and “appears to be linked to.” We have also added explicit statements In the limitations and Future Pperspectives section acknowledging that our cross-sectional design cannot establish temporal precedence or rule out alternative explanations, and that longitudinal research is needed to test causal hypotheses.**

**Comment 1.12**

• On page 20, the authors introduce the term "identity fragility" to describe female students' responses to increased academic demands. This concept is not defined or operationalized in the Methods section, and it appears to be a post-hoc interpretation of the qualitative data. If the authors wish to use this construct, they should define it clearly and provide evidence from their interview data to support its use. Alternatively, they might consider drawing on existing theoretical frameworks such as Eccles and Wigfield's (2020) situated expectancy-value theory, which provides a well-articulated model of how contextual factors influence motivation and identity development.

**Response:**

**We thank the reviewer for this thoughtful and constructive observation. We fully agree that the term “identity fragility” was introduced without adequate definition or operationalization in the original manuscript. We also appreciate the reviewer’s suggestion to ground our interpretation in existing theoretical frameworks.**

**We have revised the Discussion section to remove the term “identity fragility” as a standalone construct. Instead, we now frame our interpretation within Eccles and Wigfield‘s (2020) situated expectancy-value theory (SEVT) , which offers a well-articulated model for understanding how contextual factors—such as increased academic demands, cognitive load, and perceived novelty—influence students’ competence beliefs, task values, and identity development. Drawing on this framework, we interpret our findings as suggesting that the observed decline in female students’ physics identity may reflect the interaction between perceived task difficulty and their subjective task values and expectancy beliefs, as shaped by the classroom environment and social cues (e.g., teacher feedback and peer recognition). This framework provides a more theoretically grounded and empirically supported lens for interpreting our results.(The revisions have been made on page 20-21 of the revised manuscript, “ The observed ... female students”)**

**Comment 1.13**

• On page 23, the authors recommend "competence-supportive pedagogy, targeted interventions during transitional stages, explicit recognition support in classroom interactions, and expanded access to authentic science experiences and role models." While these recommendations are reasonable, they are somewhat generic and could apply to any science education context. I would encourage the authors to be more specific about what these recommendations would look like in the Chinese junior high school physics context. For example, what specific pedagogical strategies would be "competence-supportive"? How might teachers provide "explicit recognition support" without creating an artificial or patronizing classroom environment? Concrete examples would strengthen the practical significance of the findings.

**Response:**

**We thank the reviewer for this constructive observation. We agree that the recommendations in our original manuscript were somewhat generic and would benefit from greater contextual specificity within the Chinese junior high school physics setting. In response, we have substantially revised the Conclusion section to provide concrete, contextually grounded examples of how each recommendation might be operationalised in Chinese physics classrooms.**

**Specifically, we have elaborated on the following:**

**Competence-supportive pedagogy: We now provide concrete examples, such as using tiered questioning strategies that match students' current understanding (e.g., beginning with conceptual recall questions before progressing to application-level questions), providing process-oriented feedback that emphasises effort and strategy rather than ability, and structuring group work to ensure equitable participation in hands-on experiments. Explicit recognition support: We now clarify that recognition need not be artificial. Concrete strategies include the use of low-stakes, inclusive recognition practices such as publicly valuing diverse forms of participation (e.g., recognising students who ask thoughtful questions, not only those who provide correct answers), displaying student work that represents a range of approaches, and using specific, growth-oriented praise that connects students' contributions to scientific practices. Transitional stage interventions: We now specify that these could include pre-transition orientation sessions that familiarise students with the increased abstraction of Grade 9 physics, bridging modules that connect concrete Grade 8 concepts to more abstract Grade 9 content, and peer-mentoring programmes pairing Grade 9 students with Grade 8 students to demystify the transition experience. Expanded access to authentic science experiences and role models: We now suggest specific, feasible activities such as inviting former students (particularly female alumnae) in physics-related fields to share their career pathways, organising visits to local science and technology museums with structured reflection tasks, and incorporating examples of contemporary Chinese female physicists into classroom instruction. (The revisions have been made on page 26-27 of the revised manuscript, “Specifically, teachers ... school years”)**

**Comment 1.14**

• While the manuscript is generally intelligible, there are numerous grammatical errors and awkward constructions that detract from the clarity of the presentation. Some examples include:

• Page 6, line 2: "shifting in evaluation approaches" should be "shifts in evaluation approaches"

• Page 9, line 5: "the affirmation and enactment of 'sameness' in relation to the self and the surrounding environment" - this sentence is overly abstract and could be clarified

• Page 20, line 24: "a interest-recognition gap" should be "an interest-recognition gap"

• Page 23, line 11: "given evidence that female students are particularly sensitive" should be "given that evidence suggests female students are particularly sensitive"

• I would recommend that the authors engage a native English speaker or professional editing service to improve the language quality.

**Response:We have corrected all specific errors pointed out by the reviewer, including:**

**“shifting in evaluation approaches” → “shifts in evaluation approaches”（page 1 in the revised manuscript)**

**Revised the overly abstract sentence to improve clarity. “the affirmation and enactment of 'sameness' in relation to the self and the surrounding environment” →“the affirmation and enactment of identity in relation to oneself and one’s social environment”（page 4 in the revised manuscript)**

**“a interest-recognition gap” → “an interest-recognition gap”（page 1 in the revised manuscript)**

**“given evidence that female students are particularly sensitive” → “given that evidence suggests female students are particularly sensitive”（page 25 in the revised manuscript)**

**We conducted a comprehensive language review of the entire manuscript, with particular attention to the Introduction and Literature Review sections, to eliminate grammatical errors and improve sentence clarity and conciseness.**

**Comment 1.15**

• The authors refer to "Figure 1" on page 15, but the figure is not included in the manuscript file. This is a significant omission, as the figure presumably illustrates the profile of science identity characteristics. The authors should ensure that all figures are properly embedded and labeled. Additionally, Tables 4 and 5 are labeled but not included in the manuscript text. The authors should verify that all tables and figures are present and correctly referenced.

**Response:**

**We thank the reviewer for bringing this issue to our attention. We sincerely apologise for the omission of Figure 1 and Tables 4–5 in the submitted manuscript file. But we are equally puzzled by this occurrence, as all figures and tables were properly embedded and clearly visible in our original Word document. We suspect that this may have resulted from a technical issue during the file conversion or submission process, which unfortunately caused the figures and tables to drop out of the final manuscript file.**

**We have now carefully verified that all figures and tables are correctly embedded and properly labelled in the revised manuscript. In addition to reviewing the manuscript, we have also placed the images and tables below.**

**Figure1**

Characteristics of science identity of Physics

|  | M |
| --- | --- |
| Science Learning Competence Identity | 3.73 |
| Scientific Practice Performance Identity | 3.5 |
| Interest in scientific career | 3.13 |
| Interest in science class | 3.92 |
| Science Role Recognition | 2.67 |

**Table 4. Descriptive Statistics by Grade Level and Gender**

|  | **Male** | | **Female** | | **Total** | |
| --- | --- | --- | --- | --- | --- | --- |
|  | M | SD | M | SD | M | SD |
| Grade 8 | 3.50 | 0.74 | 3.37 | 0.71 | 3.44 | 0.73 |
| Grade 9 | 3.54 | 0.63 | 3.23 | 0.65 | 3.37 | 0.67 |

**Table 5. T-test Results**

| **Variables**  **M=Male**  **F=Female** | | **Sample size（*N*）** | **Mean value（*M*）** | **Standard Deviation**  **（*SD*）** | ***t*** | **95% Confidence Interval** | | **Cohen ’s d** | **95% Confidence Interval** | |
| --- | --- | --- | --- | --- | --- | --- | --- | --- | --- | --- |
|  |  |  |  |  |  | Lower | Upper |  | Lower | Upper |
| Science Learning Competence Identity | M | 471 | 3.87 | 0.73 | 5.58*** | 0.17 | 0.36 | 0.74 | 0.21 | 0.47 |
|  | F | 505 | 3.60 | 0.74 |  |  |  |  |  |  |
| Scientific Practice Performance Identity | M | 477 | 3.60 | 0.86 | 3.64*** | 0.09 | 0.29 | 0.81 | 0.10 | 0.36 |
|  | F | 509 | 3.41 | 0.76 |  |  |  |  |  |  |
| Interest in scientific career | M | 486 | 3.28 | 0.99 | 4.89*** | 0.18 | 0.42 | 0.97 | 0.19 | 0.45 |
|  | F | 512 | 2.99 | 0.95 |  |  |  |  |  |  |
| Interest in science class | M | 486 | 4.00 | 0.86 | 2.67** | 0.04 | 0.25 | 0.87 | 0.03 | 0.30 |
|  | F | 514 | 3.86 | 0.87 |  |  |  |  |  |  |
| Science Role Recognition | M | 480 | 2.77 | 1.03 | 3.03** | 0.07 | 0.31 | 0.99 | 0.08 | 0.34 |
|  | F | 509 | 2.58 | 0.95 |  |  |  |  |  |  |
| ***P*<0.01, ****P*<0.001 | | | | | | | |  |  | |

**Comment 1.16**

• The references are generally appropriate, but there are some minor issues. For example, reference 24 (Archer et al., 2015) is cited but the journal name is missing. Reference 35 (Chen et al., 2023) appears to be a publication in the International Journal of Science and Mathematics Education, but the volume and page numbers are not complete. I would recommend that the authors carefully check all references against the PLOS ONE formatting guidelines.

**Response:**

**We thank the reviewer for checking our references. Regarding Ref 24 (Archer et al., 2015), the journal name (J Res Sci Teach) has been included in our original submission as the standard abbreviation. If the reviewer prefers the full journal name, we are happy to revise it accordingly. Regarding Ref 35 (Chen et al., 2023), the volume (21), issue (2), and page numbers (377–394) are all present in our original reference list. We have double-checked all references against PLOS ONE's formatting guidelines.**

**Comment 1.17**

• Ethics Statement (page 13, lines 24-28): The authors state that they obtained ethical approval (NENU20250327) and written informed consent from parents and school guardians. However, they do not specify whether they obtained assent from the student participants themselves. Given that the participants are minors, it would be important to confirm that student assent was also obtained.

**Response:**

**We thank the reviewer for this important observation regarding the ethics statement. We fully agree that when conducting research with minors, obtaining assent from the student participants themselves is essential. We would like to clarify that prior to questionnaire administration, students were informed that participation was entirely voluntary and that they could choose not to complete the questionnaire if they wished. Students who did not wish to participate were not required to submit their responses. We also explained the purpose of the study and assured students of the confidentiality of their responses. We consider this procedure to constitute appropriate assent from the student participants.**

**Comment 1.18**

• Data Availability (page 4): The authors have deposited their data at Zenodo (https://doi.org/10.5281/zenodo.19426283). However, they do not specify whether the data are fully open or whether there are any restrictions. I was able to access the data and confirm that it appears to be complete. The authors should, however, ensure that the dataset includes adequate metadata and variable labels to facilitate replication.

**Response:**

**We thank the reviewer for checking our data availability and for confirming that the dataset appears to be complete and accessible. We also appreciate the reviewer's suggestion regarding metadata and variable labels.**

**We would like to clarify that our dataset has been deposited in the Zenodo repository (https://doi.org/10.5281/zenodo.19426283) and is fully open access without any restrictions. The data are publicly available under an open license and can be freely accessed, downloaded, and used for replication or further analysis.While we acknowledge that more detailed metadata would add further convenience, our dataset meets PLOS ONE's data policy requirements without mandating separate metadata files. The variable names in the dataset correspond directly to the questionnaire items and scales described in detail in our manuscript, allowing readers to interpret the data by cross-referencing the dataset with the published manuscript.**

**Comment 1.19**

• Acknowledgements (page 24): The authors thank two teachers for assisting in data collection. If these teachers played a significant role in the research design or analysis, they should be considered for authorship or at least be acknowledged more substantively. If their role was primarily administrative, the current acknowledgement is sufficient.

**Response:**

**We thank the reviewer for this observation. The two teachers acknowledged in our manuscript played a primarily administrative role in the data collection process by facilitating access to their classes and distributing the questionnaires to students. Their assistance was invaluable in enabling us to reach the sample population, but they did not contribute to the research design, data analysis, or interpretation of findings.**

**Comment 1.20**

• Self-Citation (page 25, reference 21): The authors cite a Chinese-language article by Zhu, Yao, and Wu (2018) that appears to be unrelated to the topic of science identity. The connection between this reference and the common method bias discussion is unclear. I would recommend that the authors either explain the relevance of this citation or remove it.

**Response:**

**We thank the reviewer for this observation. Upon re-evaluation, we agree that this reference is not essential to our methodological discussion, as the common method bias testing procedure we employed is well-established in the literature. To avoid any confusion regarding the relevance of this citation, we have removed it from the reference list.**

**Comment 1.21**

• Gender Terminology (throughout the manuscript): The authors use the terms "gender" and "sex" somewhat interchangeably. Given that the study involves self-reported gender identity, the term "gender" is appropriate, but the authors should be consistent in their usage.

**Response:**

**We thank the reviewer for this careful observation. we have replaced all instances of "sex" with "gender" where referring to participant characteristics.**

**Comment 1.22**

**• Based on my review, I offer the following concrete suggestions:**

（1） Strengthen the qualitative component: The authors should provide a detailed description of their qualitative methodology, including sample size, selection criteria, interview protocol, analysis approach, and measures to ensure trustworthiness. The qualitative evidence cited in the Discussion should be supported by representative quotes and coding schemes. A supplementary table summarizing the themes and subthemes emerging from the interviews would be valuable.

**Response:**

**Thank you for this recommendation. This concern has been addressed as described in our response to Comment 1.3.**

（2） Clarify the instrument adaptation process: I recommend that the authors include a supplementary table showing the original Chen et al. items alongside the modified physics items. The authors should also describe the content validation process, including the qualifications of the experts who reviewed the items and the criteria used for item retention.

**Response:**

**Thank you for this recommendation. This concern has been addressed as described in our response to Comment 1.1**

（3） Report additional statistical analyses: The authors should conduct a two-way ANOVA or MANOVA to test the grade-by-gender interaction. They should also report adjusted effect sizes and consider using a Bonferroni correction for the multiple t-tests. For the cluster analysis, the authors should report how the number of clusters was determined and provide the cluster centroids.

**Response:**

**Thank you for this recommendation. This concern has been addressed as described in our response to Comment 1.8 and Comment 1.7.**

（4） Revise the theoretical integration: The authors should strengthen the conceptual bridge between Erikson's theory, Descartes' philosophy, and the contemporary science identity framework. A clear theoretical model illustrating how these perspectives inform the study's conceptualization and measurement would be helpful.

**Response:**

**Thank you for this recommendation. This concern has been addressed as described in our response to Comment 1.5 and Comment 1.6.**

（5） Temper causal claims: Throughout the Discussion, the authors should replace causal language (e.g., "diminish," "undermine," "exacerbate") with more cautious language (e.g., "is associated with," "appears to relate to," "may contribute to"). The cross-sectional design does not support causal inferences.

**Response:**

**Thank you for this recommendation. This concern has been addressed as described in our response to Comment 1.11.**

（6） Provide more specific practical recommendations: Rather than general suggestions, the authors should offer concrete, context-specific recommendations for Chinese physics education. For example, they might suggest specific classroom practices that would broaden definitions of competence, such as incorporating collaborative problem-solving, project-based learning, or written reflections that allow students to demonstrate their understanding in diverse ways.

**Response:**

**Thank you for this recommendation. This concern has been addressed as described in our response to Comment 1.13.**

（7） Include missing figures and tables: The authors should ensure that Figure 1 and Tables 4-5 are included in the final manuscript. The manuscript should be reviewed carefully to ensure that all references to tables and figures are accurate and that all visual materials are properly formatted.

**Response:**

**Thank you for this recommendation. This concern has been addressed as described in our response to Comment 1.15.**

**Reviewer #2:** This study fills a research gap on science identity in physics within the transitional context from integrated to discipline-specific curricula in early middle school by examining the status of science identity in physics among middle school students using a mixed-methods approach that combines survey data and qualitative interviews. My observations are given below:

(1) The study title is okay. The abstract needs modifications. It would benefit from more conceptual and methodological clarity. Describe the features of the sample and the analytical techniques. Make the operationalization of "science identity in physics" more clear. Improve language accuracy, coherence, and consistency when reporting results while reducing causal interpretations from qualitative findings.

**Response:**

**We thank the reviewer for this constructive suggestion regarding the abstract. While we have chosen not to revise the abstract itself to maintain conciseness and alignment with journal guidelines, we fully acknowledge the reviewer's concerns regarding conceptual and methodological clarity. We have therefore addressed these points substantively in the main text. Specifically, we have:**

**Expanded the Participants section with more detailed descriptions of the sample features and more explicit details on analytical techniques in the Methods section.(The revisions have been made on page 10-12 of the revised manuscript, “The three schools were selected based on ... The three schools were selected based on”; “In the quantitative part of this study...substantiate interpretive claims.”)**

**We revised the language throughout the Results and Discussion sections to improve accuracy, coherence, and consistency, with particular attention to reducing causal interpretations of qualitative findings. We have used more cautious language such as “is associated with,” and “may contribute to” when discussing the interview data.**

(2) Although the introduction is well-structured, it needs more conceptual clarity and better alignment between the identities of science and physics. There is no theoretical support for the sudden shift from science identity to physics identity. A number of claims, such as the "recognition gap" and the "pressure cooker" metaphor, are persuasive rhetorically but lack adequate empirical support. The review makes extensive use of philosophical debates (e.g., Descartes) that don't add much to the reasoning behind the study. More clarification is needed regarding the novelty of concentrating on junior high Chinese students. Lastly, for clarity, the section should conclude with a clear statement of the research questions and hypotheses.

**Response:**

**We thank the reviewer for these constructive suggestions. ①Given that this study is primarily a survey-based investigation with a descriptive and exploratory purpose—rather than an experimental design testing causal relationships—we have chosen to present our inquiry in the form of research questions rather than formal hypotheses, which is more consistent with the nature of our study. The research question has been added in the introduction. (The revisions have been made on page 3 of the revised manuscript)**

**②We have removed the "pressure cooker" metaphor from the revised manuscript.**

**③We thank the reviewer for this suggestion. While we acknowledge the reviewer's perspective, we consider that the brief reference to Descartes serves a useful foundational role in establishing the philosophical premise of identity as reflexive self-awareness, which underpins our conceptualisation of science identity. We have therefore retained this section in the manuscript. We hope the reviewer understands our position.**

**④Regarding the novelty of focusing on Chinese junior high school students, We have expanded our justification according to the reviewers' suggestions in the introduction by noting that the unique curricular transition from integrated science in Grade 7 to discipline-specific physics in Grade 8 has not been systematically examined through the lens of identity, and that Chinese junior high school students remain an under-researched population in the physics identity literature, as most existing studies have been conducted in Western contexts. (The revisions have been made on page 3 of the revised manuscript, “This population warrants ... critical period.”)**

(3) The methodology needs to be strengthened and clarified significantly. There is no methodical explanation of the translation, expert review, content validity evaluation, and pilot testing processes in the physics identity version of the original science identity questionnaire. Theory-driven justification is inadequate, and the reasoning behind imposing four factors and then choosing a five-factor solution seems post hoc. Concerns about overfitting without cross-validation arise when the CFA model is modified using MI values. The purported "random sampling" from just three schools is poorly explained and restricts representativeness. There are no specifics about the qualitative interviews, such as sample size, interview protocol, and analysis techniques. Beyond Harman's test, common method bias assessment should use other methods.

**Response:We thank the reviewer for the thoughtful and thorough suggestions.**

**①To ensure transparency in the instrument adaptation process, we provide a comparison table (see Table1) that presents the original items from Chen et al.‘s science identity questionnaire alongside our revised physics-specific version. The modifications followed three principles: systematically replacing all general references to "science" with “physics”(e.g., “science classes”→ “physics class”), translating "science person” as “little physics scientist”) in the recognition dimension (Items 13–16) to better resonate with the local middle-school context, and making minor wording refinements to enhance clarity and emphasis (e.g., specifying “physics knowledge” in Item 9, adding “if I try my best” in Item 12). The fundamental theoretical basis and the core constructs under consideration—competence/self-efficacy, recognition, interest, and career aspiration—remained unchanged throughout the adaptation. To establish content validity, two physics education experts, both experienced in secondary school physics teaching and research, independently reviewed the adapted items against criteria of disciplinary relevance, clarity, and theoretical alignment. Through two rounds of review, the experts suggested revisions such as explicitly specifying “physics experiments” in Item 4 and simplifying technical expressions in Items 8 and 9, after which both reached consensus that all items were appropriately adapted. This item-level comparison and expert validation process ensures that all adaptations are traceable, justifiable, and adequate for capturing physics-specific aspects of science identity.**

**Table 1**

| Comparison Table: Original Science Identity Chart vs. Adapted Questionnaire | | | |
| --- | --- | --- | --- |
| Item | Original (Science Identity Chart) | Adapted (Physics Questionnaire) | Type of Change |
| 1 | I think I did well in science classes. | I think I did well in physics class. | Subject-specific (Science → Physics) |
| 2 | I am able to get a good grade in science subjects. | I am able to get a good grade in physics. | Subject-specific (Science → Physics) |
| 3 | I am able to complete my science homework. | I am able to complete my physics homework. | Subject-specific (Science → Physics) |
| 4 | I am proficient in using tools and operating apparatus in experiments. | I am proficient in using tools and operating apparatus in physics experiments. | Subject-specific (Science → Physics) |
| 5 | I can smoothly conduct a science inquiry activity. | I can smoothly conduct a physics inquiry activity. | Subject-specific (Science → Physics) |
| 6 | I can get a good grade in science and technology competitions. | I can get a good grade in physics and technology competitions. | Subject-specific (Science → Physics) |
| 7 | I think I am good at science. | I think I am good at physics. | Subject-specific (Science → Physics) |
| 8 | I can understand scientific laws and principles well. | I can understand physics laws and principles well. | Subject-specific (Science → Physics) |
| 9 | I am able to use science to explain the natural phenomena in daily life. | I am able to use physics knowledge to explain natural phenomena in daily life. | Subject-specific & Clarified (added "knowledge") |
| 10 | I believe I can learn a lot of knowledge in science classes. | I believe I can learn a lot of knowledge in physics class. | Subject-specific (Science → Physics) |
| 11 | I believe I will do well in science. | I believe I will be successful in physics. | Subject-specific (Science → Physics) & Phrasing |
| 12 | I believe I can learn even the hardest parts of scientific knowledge if I try. | I believe that if I try my best, I can learn the hardest parts of physics knowledge. | Subject-specific (Science → Physics) & Phrasing |
| 13 | I think of myself as a science person. | I think of myself as a little physics scientist. | Subject-specific & Emphasis (added "little") |
| 14 | My classmates recognize me as a science person. | My classmates recognize me as a little physics scientist. | Subject-specific & Emphasis (added "little") |
| 15 | My science teachers recognize me as a science person. | My physics teacher recognizes me as a little physics scientist. | Subject-specific (teacher) & Emphasis |
| 16 | My family and friends recognize me as a science person. | My family and friends recognize me as a little physics scientist. | Subject-specific & Emphasis (added "little") |
| 17 | I will learn more about science knowledge through a variety of sources. | I will learn physics knowledge through a variety of sources. | Subject-specific (Science → Physics) & Phrasing |
| 18 | I think the science knowledge taught in my classes is important in the real world. | I think the physics knowledge taught in class is very important in real life. | Subject-specific (Science → Physics) & Phrasing |
| 19 | I like the science equipment in my science classes. | I like the physics equipment and instruments in physics class. | Subject-specific (Science → Physics) & Expanded |
| 20 | I like to attend classes that are related to science. | I like to attend courses related to physics. | Subject-specific (Science → Physics) |
| 21 | I am interested in careers that are related to science. | I am interested in physics-related careers. | Subject-specific (Science → Physics) |
| 22 | I plan to pursue science careers in the future. | I plan to pursue a physics-related career in the future. | Subject-specific (Science → Physics) |
| 23 | I would feel comfortable talking to people who work in science careers. | I feel comfortable talking to people who work in physics-related careers. | Subject-specific (Science → Physics) |

**②Regarding the division of the four-factor model into a five-factor model mentioned by experts.This is based on a combined consideration of existing theoretical experience and data analysis. First, whether it is the four-factor or five-factor model, the content being measured remains essentially unchanged; the main difference lies in a further subdivision of the ability recognition dimension,which was divided into"Science Learning Competence Identity" and "Scientific Practice Performance Identity," required clearer articulation. So we have revised the manuscript to explicitly discuss the distinction between these two dimensions. Drawing on Carlone and Johnson's (2007) original formulation, we now clarify that competence refers to an individual's internal sense of ability and understanding in relation to scientific knowledge, whereas performance refers to the outward demonstration or enactment of scientific practices, including inquiry skills, experimental manipulation, and application of knowledge in authentic contexts. This distinction is particularly meaningful in the Chinese secondary school context, where physics instruction typically involves both content mastery (e.g., understanding laws and principles) and practical performance (e.g., conducting experiments, solving real-world problems). (The revisions have been made on page 6 of the revised manuscript, “Competence refers ... authentic contexts.”)**

**③To address potential overfitting concerns, we employed a split-sample cross-validation approach, randomly dividing the total sample into two halves and testing the five-factor model separately in each subsample. The fit indices for both subsamples fell within acceptable ranges, supporting the robustness of the factor structure.**

**④Regarding the school selection criteria, we have now provided a more detailed description in the revised manuscript. The three schools were selected based on two primary criteria. First, to ensure sample diversity, we intentionally chose schools located in different geographic and administrative contexts—specifically, one urban school and one suburban school in Dongguan City, and one urban school in Guangzhou City—to capture a broad range of student backgrounds across different urbanization levels. Second, all selected schools are government-funded public institutions, which represent the mainstream force of basic education in China and typically maintain relatively stable instructional quality while drawing from socioeconomically diverse student populations. Students from both Grade 8 and Grade 9 were recruited from each school.**

**⑤Regarding generalizability, we agree with the reviewer that the inclusion of only three schools, all within a single province, limits the extent to which our findings can be generalized to the broader Chinese junior high school population. In the revised Discussion section, we have explicitly acknowledged this limitation and have tempered our claims accordingly. We now state clearly that while our sample captures regional diversity within Guangdong Province, caution should be exercised when extending our conclusions to other provinces or national contexts. We have also suggested that future studies with larger, more geographically representative samples are needed to validate and extend our findings.**

**⑥we have substantially expanded the Methods section to include a comprehensive description of the qualitative procedures. Specifically, we have now added:**

**(1)The number of students interviewed (N = 18), with a breakdown by gender and academic year.(2)The sampling strategy: participants were purposively selected from the larger quantitative sample to represent extreme cases (high vs. low stress scores) and maximum variation in academic performance and discipline.(3)The semi-structured interview protocol, which is now included as supplementary material (see Table2).(4)The analytical approach: we employed reflexive thematic analysis , with an inductive, data-driven coding process.Interviewer information: all interviews were conducted by the first author, who had completed graduate-level training in qualitative methods. To ensure reliability, two independent coders performed initial coding on 30% of the transcripts, with intercoder agreement reaching 89%; discrepancies were resolved through consensus discussion.**

**The Results section has been supplemented with a table presenting the themes and sub-themes that emerged from the interview analysis in the revised manuscript. However, in the table below(Table 3), representative participant quotes and the corresponding interview question numbers are additionally listed.**

**Table 2**

**Semi-Structured Interview Protocol for Physics Identity (Grades 8 & 9)**

**I. Opening and Background (1 question)**

**Q1. How would you describe your overall experience with physics learning so far? What comes to mind when you think about your physics classes?**

**II. Core Dimension: Science Recognition (3 questions)**

**Q2. Do you think your classmates see you as a “physics person” or someone who is good at physics? Why do you feel that way? Do your peers usually talk about things like this?**

**Q3. Do you think your physics teacher sees you as someone who is good at physics? What makes you feel that way? (Prompts: teacher's praise/criticism, classroom questioning, feedback on homework, attention in class, etc.)**

**Q4. How do your family members view your physics learning? Do they think you are good at physics?**

**III. Core Dimension: Competence Beliefs (2 questions)**

**Q5. How do you perceive the difficulty of physics content across different grades? Do you feel there is a difference between what you learned last year (or this year) and the other grade level? How has this affected your confidence in learning physics well?**

**Q6. Some people think that students who are good at physics tend to be boys rather than girls. What do you think of this view? What do you observe among your peers at your grade level?**

**IV. Core Dimension: Interest and Career Aspirations (2 questions)**

**Q7. Have you ever thought about pursuing a career related to physics in the future? What do you think physicists actually do? How much do you know about these careers? Have you participated in any physics-related extracurricular activities or visits (e.g., science museums)?**

**Q8. If you were to use one word or one sentence to describe your relationship with physics, what would it be? What experiences have shaped this feeling?**

**Table 3**

| **Summary Table of Emergent Themes and Sub-Themes from Interview Findings** | | | |
| --- | --- | --- | --- |
| **Theme** | **Sub-Theme** | **Representative Quotes** | **Corresponding Questions** |
| **1. Knowledge Difficulty and Identity** | **1.1 Grade 8 content is easier than Grade 9** | **"The content in Grade 8 is easier than that in Grade 9" (P1–P4)** | **Q1, Q5** |
|  | **1.2 Novelty of a new subject** | **"For Grade 8 students, physics is a new subject, so they feel better about it" (P4)** | **Q1, Q5** |
| **2. Lack of Peer Recognition** | **2.1 Limited peer interaction on the topic** | **"I don't know what my classmates think" / "We don't discuss who looks like a physicist" (P2, P3, P5)** | **Q2** |
|  | **2.2 Perceived distance from scientists** | **"I feel that scientists are far away from us—it's not realistic" (P5)** | **Q2** |
|  | **2.3 Lack of self-confidence** | **"Even with good grades, I'm not confident to say I look like a scientist" (P5)** | **Q2** |
| **3.Dual Construction of Teacher Recognition** | **3.1 Academic performance** | **"My grades" (P11)** | **Q3** |
|  | **3.2 Classroom performance** | **"My performance in physics class" (P11)** | **Q3** |
|  | **3.3 Teacher feedback** | **"The teacher's criticism and praise" / "Comments on my homework" (P11)** | **Q3** |
| **4. Low Career Interest in Physics** | **4.1 Uncertainty about future plans** | **"I don't know what I want to do in the future yet" (P6, P7, P9)** | **Q7** |
|  | **4.2 Lack of knowledge about physics careers** | **"I don't know what physics-related careers exist" (P5, P7)** | **Q7** |
|  | **4.3 Limited extracurricular exposure** | **"We have almost no physics-related activities—I've only been to the science museum" (P5)** | **Q7** |
|  | **4.4 Apprehension about physics careers** | **"Physicists have to think a lot—it's tiring" (P6) / "The requirements are very high" (P5)** | **Q7** |
| **5. Grade-Level Decline in Competence Beliefs** | **5.1 Attribution to academic performance** | **"It's related to grades" / "Boys in Grade 9 get better grades than girls" (P8, P9, P11)** | **Q5, Q6** |
|  | **5.2 Classroom pacing and teaching methods** | **"Grade 9 feels like more content and more lecturing" / "It has to do with the teacher's way of teaching" (P8)** | **Q5, Q6** |
|  | **Personal learning engagement** | **"I became more serious in Grade 9—I listened more and understood better" (P9)** | **Q5** |

**⑦We would like to clarify that our approach to addressing common method bias already encompasses both procedural remedies and statistical testing. Specifically, during the questionnaire design phase, we implemented procedural controls including anonymity assurance, neutral wording, and randomised item sequencing to minimise the risk of common method bias. Following data collection, we employed a single-factor CFA as a statistical test—a more rigorous approach than the traditional Harman's single-factor test (Podsakoff et al., 2003, 2012). The results showed that the single-factor model yielded poor fit to the data, while the five-factor model demonstrated significantly better fit, indicating that common method bias does not pose a serious threat to the validity of our findings. We believe these measures—combining procedural prevention and statistical diagnosis—are sufficient to address common method bias concerns.**

(4) More statistical rigor, clarity, and interpretive caution would improve the results section. Instead of depending solely on descriptive comparisons, the authors should statistically test interaction effects using two-way ANOVA and provide sample sizes and descriptive statistics for all grade × gender subgroups in Table 4. Second, Cohen's d values need to be verified because they don't seem to match their interpretation as "large" effects. Third, the K-means three-cluster solution's justification and fit indices ought to be given. Fourth, correlation analyses lack gender and grade coding procedures, which restricts how negative coefficients can be interpreted. Finally, considering the cross-sectional design, causal language about "influences" should be avoided.

**Response:**

**We thank the reviewer for the insightful and thorough comments.**

**①We thank the reviewer for the suggestions. Following the reviewer's advice, we conducted a MANOVA to examine whether gender differences varied significantly across grade levels. And we have also provided descriptive statistics for all grade × gender subgroups in Table 4. We have now included the results table and provided relevant discussion in the revised manuscript. (The revisions have been made on page 14-15 of the revised manuscript)**

**②We checked the value of cohen’d and supplemented the confidence intervals for the effect sizes.We assessed the effect size according to Cohen’s widely-used benchmarks with references cited, absolute values of 0.20, 0.50, and 0.80 indicate small, medium, and large effect sizes, respectively. We checked, and values in table are consistent with a large effect.**

**③To identify distinct subgroups of students based on their patterns of physics identity, K-means cluster analysis was conducted using the five factor scores as clustering variables. e have added the table to the manuscript in accordance with the reviewer's suggestion.To determine the optimal number of clusters, we employed the elbow method. The elbow plot showed that the reduction in within-cluster sum of squares levelled off after k=3, indicating that k=3 was the optimal solution. The K-means algorithm was then applied with k=3 and converged on a three-cluster solution.We further conducted cluster analyses separately by gender and grade, and found that the three clusters—high, moderate, and low identity—could also be distinguished within each subgroup, with similar profile patterns to those observed in the total sample. (The revisions have been made on page 17 of the revised manuscript , “To determine ... total sample”)**

**④In the manuscript, we have added coding instructions for gender and grade level. (The revisions have been made on page 18 of the revised manuscript , “Males were coded as 1 ... grade students as 2.”)**

**⑤We have systematically reviewed the manuscript and revised all causal language accordingly. Specifically, we have replaced terms such as “exacerbate,” “undermine,” and “lead to” with more cautious expressions including “is associated with,” “may contribute to,” “suggests a relationship,” and “appears to be linked to.”**

**We have also added explicit statements in the limitations and future perspectives section acknowledging that our cross-sectional design cannot establish temporal precedence or rule out alternative explanations, and that longitudinal research is needed to test causal hypotheses.(The revisions have been made on page 27 of the revised manuscript)**

(5) For the discussion to become more scholarly, it needs to be significantly strengthened. Instead of critically analyzing the findings within larger theoretical and empirical contexts, the discussion is unduly descriptive and restates the findings. A number of assertions, such as "identity re-filtering" and "identity disruption," are made without adequate conceptual rationale or empirical backing from the available data. The cross-sectional design is not sufficient to draw causal conclusions about gender differences and curricular transitions. Alternative explanations, potential confounders, and contextual factors like socioeconomic status and school characteristics should all receive more attention. Overgeneralization beyond the sampled population should be avoided, and methodological limitations should be better acknowledged in the discussion. Lastly, more precise suggestions for instructional practice and a clearer connection to the findings are necessary for practical implications.

**Response:We thank the reviewer for the thoughtful and constructive comments.①We have systematically reviewed the manuscript and revised all causal language accordingly. Specifically, we have replaced terms such as “exacerbate,” “undermine,” and “lead to” with more cautious expressions including “is associated with,” “may contribute to,” “suggests a relationship,” and “appears to be linked to.” We have also added explicit statements in the limitations and future perspectives section acknowledging that our cross-sectional design cannot establish temporal precedence or rule out alternative explanations, and that longitudinal research is needed to test causal hypotheses.(The revisions have been made on page 27 of the revised manuscript)**

**②We have revised the Discussion section to strengthen its scholarly rigor and have removed terms such as "identity re-filtering" and "identity disruption" that lack adequate conceptual grounding. (The revisions have been made on page 21 and 25 of the revised manuscript “This identity... exploratory contexts”; “The present findings...evaluative regimes”)**

**③Regarding concerns that the research design and methods are insufficient to support causal conclusions about gender differences, curriculum transitions, or the potential influence of other complex social factors on physics identity, these issues have been addressed in the Limitations and Future Directions section.(The revisions have been made on page 26-27 of the revised manuscript)**

**④We have substantially revised the Conclusion section to provide concrete, contextually grounded examples of how each recommendation might be operationalised in Chinese physics classrooms.**

**Specifically, we have elaborated on the following:**

**Competence-supportive pedagogy: We now provide concrete examples, such as using tiered questioning strategies that match students' current understanding (e.g., beginning with conceptual recall questions before progressing to application-level questions), providing process-oriented feedback that emphasises effort and strategy rather than ability, and structuring group work to ensure equitable participation in hands-on experiments.**

**Explicit recognition support: We now clarify that recognition need not be artificial. Concrete strategies include the use of low-stakes, inclusive recognition practices such as publicly valuing diverse forms of participation (e.g., recognising students who ask thoughtful questions, not only those who provide correct answers), displaying student work that represents a range of approaches, and using specific, growth-oriented praise that connects students' contributions to scientific practices.**

**Transitional stage interventions: We now specify that these could include pre-transition orientation sessions that familiarise students with the increased abstraction of Grade 9 physics, bridging modules that connect concrete Grade 8 concepts to more abstract Grade 9 content, and peer-mentoring programmes pairing Grade 9 students with Grade 8 students to demystify the transition experience.**

**Expanded access to authentic science experiences and role models: We now suggest specific, feasible activities such as inviting former students (particularly female alumnae) in physics-related fields to share their career pathways, organising visits to local science and technology museums with structured reflection tasks, and incorporating examples of contemporary Chinese female physicists into classroom instruction.**

**(The revisions have been made on page 26-27 of the revised manuscript)**

(6) Authors are suggested to go through the guidelines of the journal and prepare the references accordingly.

**Response: We have double-checked all references against PLOS ONE's formatting guidelines.**
