# Supplementary material for: A study on the status quo and influencing factors of science identity in physics among junior high school students
Source: PLoS One. 2026 Oct 13;21(10):e0358695. doi: 10.1371/journal.pone.0358695 (PMC13660884; doi:10.1371/journal.pone.0358695)
Supplement: Supplementary file 2 [file pone.0358695.s003.docx]

**Response to Reviewers**

We sincerely thank the academic editor and the reviewers for their time, expertise, and constructive feedback on our manuscript. We have carefully considered all comments and have revised the manuscript accordingly. Below we provide our point-by-point responses to each comment. All changes in the revised manuscript have been marked using Track Changes.

Reviewer #1: Remaining Minor Concerns

We are grateful to the reviewer1 for the positive evaluation and for acknowledging that all previous comments have been addressed. We also appreciate the two remaining minor concerns, which we have carefully considered and responded to below.

1. Abstract modifications: While the authors chose not to revise the abstract to maintain conciseness, some of the conceptual and methodological clarity issues raised by Reviewer #2 could have been addressed with minor abstract revisions. However, the main text now adequately covers these points.

Response:We thank the reviewer for this suggestion. In response, we have briefly incorporated a statement regarding the research methods into the Abstract, while maintaining conciseness as required by the journal. (Page 1, “...Quantitative analyses, including descriptive statistics, cluster analysis, correlation analysis, t-tests, and MANOVA, examined students’ science identity status, patterns, and gender differences. Qualitative interviews were analyzed using reflexive thematic analysis to explore underlying factors.”)

2. School-level differences: Although the authors acknowledge the nested nature of the data and recommend multilevel modeling for future research, the current analyses treat all participants as a single analytical sample. This is acceptable given the exploratory purpose and small number of schools, but it remains a limitation worth emphasizing.

Response:We appreciate the reviewer’s thoughtful comment. In response, we have explicitly emphasized the nested nature of the data in the Limitations and Future Directions section, highlighting it as a limitation of primary and particular concern. Please see page 27 for the specific changes. “ First, and of particular note, school-level differences were not explicitly controlled for in the primary analyses, as the principal objective was to examine overarching patterns rather than to conduct cross-school comparisons. Given the inherent nested structure of the data (students clustered within schools) and the limited number of schools involved, this limitation warrants particular consideration. Furthermore, in light of this nested structure, future investigations employing multilevel modeling with a larger sample of schools are recommended to more rigorously assess the effects of school-level variables on students’ science identity.”

Reviewer #2: Well done, authors. I thoroughly examined the revised manuscript and found that the authors had successfully incorporated the recommendations from the earlier review. It does not require further changes.

Response: We would like to express our sincere gratitude to the reviewer for the highly positive and encouraging evaluation of our revised manuscript. We greatly appreciate the careful examination of the revised manuscript and the recognition that we have successfully addressed the recommendations raised during the previous round of review. We are deeply grateful for the reviewer’s time, patience, and constructive feedback throughout the review process.
